# Genomic insights into *HSFs* as candidate genes for high-temperature stress adaptation and gene editing with minimal off-target effects in flax

**DOI:** 10.1038/s41598-019-41936-1

**Published:** 2019-04-03

**Authors:** Dipnarayan Saha, Pranit Mukherjee, Sourav Dutta, Kanti Meena, Surja Kumar Sarkar, Asit Baran Mandal, Tapash Dasgupta, Jiban Mitra

**Affiliations:** 1 Division of Crop Improvement, ICAR-Central Research Institute for Jute and Allied Fibres, Kolkata, West Bengal 700121 India; 2 Faculty Centre for Integrated Rural Development and Management, Ramakrishna Mission Vivekananda Educational and Research Institute, Ramakrishna Mission Ashrama, Narendrapur, Kolkata, 700103 West Bengal India

## Abstract

Flax (*Linum usitatissimum*) is a cool season crop commercially cultivated for seed oil and stem fibre production. A comprehensive characterization of the *heat shock factor* (*HSF*) candidate genes in flax can accelerate genetic improvement and adaptive breeding for high temperature stress tolerance. We report the genome-wide identification of 34 putative *HSF* genes from the flax genome, which we mapped on 14 of the 15 chromosomes. Through comparative homology analysis, we classified these genes into three broad groups, and sub-groups. The arrangement of HSF-specific protein motifs, DNA-binding domain (DBD) and hydrophobic heptad repeat (HR-A/B), and exon-intron boundaries substantiated the phylogenetic separation of these genes. Orthologous relationships and evolutionary analysis revealed that the co-evolution of the *LusHSF* genes was due to recent genome duplication events. Digital and RT-qPCR analyses provided significant evidence of the differential expression of the *LusHSF* genes in various tissues, at various developmental stages, and in response to high-temperature stress. The co-localization of diverse cis-acting elements in the promoters of the *LusHSF* genes further emphasized their regulatory roles in the abiotic stress response. We further confirmed DNA-binding sites on the LusHSF proteins and designed guide RNA sequences for gene editing with minimal off-target effects. These results will hasten functional investigations of *LusHSFs* or assist in devising genome engineering strategies to develop high-temperature stress tolerant flax cultivars.

## Introduction

The impact of global warming on crop productivity is alarming and presumed to decrease global crop yield by 1.5% per decade. The effects of high-temperature (HT) stress are detrimental to plant growth and development, physiological processes, and crop yield per se^1^. At the cellular level, basic stresses, such as temperature, drought, and salinity, result in cell injury due to osmotic and oxidative stresses. Being immobile, plants respond through a variety of adaptive, avoidance, and/or acclimation mechanisms to mitigate HT stress. These responses include the activation of various physiological and biochemical processes, antioxidant defences, and metabolite synthesis pathways. Similarly, several genetic components, such as structural and regulatory genes perform essential roles in HT stress alleviation.

Heat shock proteins (HSPs) are molecular chaperones that execute crucial functions in response to HT stress. These proteins respond by the folding, accumulation, and degradation of other protective proteins when cells are exposed to HT stress^2^. The expression of these HSP-coding genes is regulated by a group of DNA-binding transcription factors, known as heat shock factors (HSFs). Therefore, HSFs are the primary regulators of the HT stress-responsive gene expression pathway, which operates by modulating a cascade of signal transduction networks^3^. Structurally, HSF proteins comprise an N-terminal conserved DNA-binding domain (DBD) of helix-turn-helix motifs that specifically interact with the *heat shock elements* (*HSEs*) of *HSP* gene promoters. Adjacent to DBD exists the oligomerization domain (OD) with the characteristic heptad hydrophobic repeat (HR-A/B) motif. Variations in the amino acid residues of HR-A/B motifs and the distance between the DBD and the OD facilitate the grouping of HSF proteins. Plant HSFs are grouped together within HSFA, B, and C with further sub-groups existing within the respective groups. In addition, HSF proteins also comprise a C-terminal activation domain (CTAD) with an AHA motif, often nuclear localization (NLS) and nuclear export signals (NES)^4^. The intra-HSF protein domain interactions usually regulate the activation and cellular localization of HSF proteins. Under natural conditions, the HSFA monomer, containing one C-terminal and three N-terminal leucine zipper repeats, is suppressed by an association with HSPs to inactivate this protein in the cytosol. A bi-partite NLS sequence flanking the N-terminal zippers confers nuclear localization. An interaction between the N- and C-terminal zippers in the HSFA monomer masks the NLS sequence. In addition, the interaction of HR-A/B motifs maintains HSFA in a monomeric form and negatively regulates CTAD under normal conditions. Upon HT stress, several proteins in the cell misfold, to which HSPs interact and become dissociated from HSFA. This dissociation allows HSFA to form trimers, expose the NLS sequence and translocate to the nucleus to trigger transcription. The DBD of the trimeric HSF recognizes at least three copies of a typical penta-nucleotide sequence, 5′-*nGAAn*-3′, in the *HSE* to regulate *HSP* transcription^3^. With much sequenced plant genome data available, a large number of *HSFs* were characterized in several plant species, and their putative roles were predicted through gene expression studies^3^. The genome-wide analysis of *HSF* genes in various plants has revealed their regulatory roles not only in HT stress but also in other abiotic stress responses. This finding emphasizes their possible involvement in a complex crosstalk among the different stress response pathways^4^. Hence, *HSFs* are excellent candidate genes for genetic engineering, gene editing or breeding of climate-resilient crops. A thorough delineation of these key factors at the genome-scale is indispensable to the target species before they can be harnessed in any genetic improvement programme.

Flax (*Linum usitatissimum* L.) is an important global cash crop producing seed oil (linseed) and bast tissue-derived fibre (linen) as economic products. For various reasons, there is a renewed interest in the cultivation and advanced scientific study of flax. Seed oil from flax is an abundant source of alpha-linolenic acid (ALA) and omega-3 fatty acid. It serves as an exceptional food, feed and industrial feedstock for several purposes. In addition, the cellulosic stem fibre serves as a source for fine textile-grade fabric, the geotextile industry, the composite industry, and the paper and pulp industry^5,6^. Since flax is a rabi season crop, HT stress is one of the major limiting factors of flax cultivation, especially at the terminal stages. A cold temperature over an extended period is essential for fibre maturation. Thus, the adaptability of elite fibre flax genotypes to warmer climates is extremely poor. The HT stress of 40 °C, over five to seven days, affects pollen viability, boll formation, and seed setting^7,8^. However, the genetically variable superior alleles of *HSPs* and *HSFs* can be harnessed to breed flax varieties with an enhanced capacity to adapt to warm climatic conditions. A comprehensive analysis of the *HSF* genes from the flax genome^9^ is thus apt for the genetic improvement of flax with an enhanced resilience to adverse climatic conditions. In this study, we identified and characterized *HSFs* from the flax genome. The characterization included phylogeny, evolutionary time, and gene expression analysis in tissues and with HT stress treatment. We also identified *guide RNA* (*gRNA*) sequences from the *LusHSFs* to be used in functional studies and genetic improvement through gene editing with the aim of obtaining minimal off-target genomic effects.

## Results

### *HSF* gene identification in the flax genome and their sequence features

A search for HSFs Hidden Markov Model (HMM)-based Pfam ID PF00447 in the genome of *L*. *usitatissimum* (cv. CDC Bethune) hosted in the Phytozome database produced 40 sequences. The individual HSF protein sequences were further supported by scanning against the Pfam-A database at the E-value threshold of 10^−3^ and the Simple Modular Architecture Research Tool (SMART) web server for the presence of the characteristic HSF-DBD and coiled-coil structures. Finally, the 40 putative HSF protein sequences were analysed in the HEATSTER database, revealing six loci (Lus10005925, Lus10016634, Lus10022546, Lus10026819, Lus10029852, and Lus10038874) consisting of incomplete domains that are essential for classification as HSF proteins (Supplementary Table S1). These six loci were removed from further analysis because they lacked the essential ‘coiled coil’ oligomeric domain (HR-A/B region), which functions through trimerization upon HT response. The remaining 34 HSF sequences consisting of characteristic DBD, HR-A, and HR-B motifs were named LushsfA1a to LushsfC1b based on their classification in the HEATSTER database (Table 1). Other domains, such as NLS, NES, activator motifs (AHA), and tetrapeptide repressor domain (RD), were also located on the LusHSF proteins. As per Table 1, the length of the *LusHSF* genes and their CDS ranged from 912 bp (*LushsfA4c*) to 3585 bp (*LushsfA1d*) and 273 bp (*LushsfB5b*) to 1473 bp (*LushsfA4d*), respectively. The amino acid sequence length of the LusHSF proteins varied from 200 (LushsfB5b) to 822 (LushsfB1a) amino acids. The molecular weight (Mw) and isoelectric points (pI) of the LusHSF proteins ranged from 23.19 (LushsfB5b) to 55.15 (LushsfA4d) kDa and 4.78 (LushsfA8b) to 9.32 (LushsfB5a), respectively. The GRAVY score of each LusHSF protein was found to be negative, ranging from −0.995 to −0.499, indicating that these proteins are highly polar molecules. Subcellular localization predictions of the LusHSF proteins based on the k-nearest neighbour classifier of the WoLF PSORT program showed that most of these proteins are localized in the nucleus.

**Table 1:** Features of *LusHSF* genes and proteins in the flax genome.

HSF ID	Phytozome	HSF	Location			Strand	Length			pI	GRAVY	Mw (kD)	Subcel loc and no. NN
	locus	group	Chr*	Start*	End*		Gene	CDS	Protein				
*LushsfA1a*	Lus10011065	A1a	Lu08	18101211	18103152	reverse	1941	1377	458	4.82	−0.578	50.06	nu: 14
*LushsfA1b*	Lus10040911	A1b	Lu15	8448889	8450468	forward	1579	1377	458	5.65	−0.583	50.64	nu: 14
*LushsfA1c*	Lus10000312	A1c	Lu08	16643985	16645926	reverse	1941	1377	458	4.95	−0.602	50.01	nu: 14
*LushsfA1d*	Lus10030956	A1d	Lu09	5719143	5722728	reverse	3585	1416	471	4.90	−0.778	52.43	nu: 14
*LushsfA1f*	Lus10040091	A1f	Lu07	4032070	4035485	forward	3415	1416	471	4.72	−0.770	52.11	nu: 14
*LushsfA2a*	Lus10013797	A2a	Lu11	2671577	2672762	reverse	1185	1026	341	5.01	−0.682	38.43	nu: 14
*LushsfA2b*	Lus10039134	A2b	Lu14	18116147	18117412	forward	1265	1110	369	5.22	−0.649	41.46	nu: 13, cp: 1
*LushsfA3a*	Lus10023866	A3a	Lu08	4723718	4725601	forward	1883	1326	441	4.73	−0.570	49.81	nu: 14
*LushsfA3b*	Lus10014369	A3b	Lu06	14835072	14836968	forward	1896	1341	446	4.71	−0.561	50.31	nu: 14
*LushsfA4a*	Lus10007318	A4a	Lu15	2083596	2084949	reverse	1353	1242	413	4.97	−0.648	46.38	nu: 14
*LushsfA4b*	Lus10015237	A4b	Lu12	2940951	2942258	reverse	1307	1212	403	5.75	−0.880	45.68	nu: 14
*LushsfA4c*	Lus10029269	A4c	Lu03	8165193	8166105	reverse	912	801	266	6.01	−0.623	30.58	nu: 13, cp: 1
*LushsfA4d*	Lus10005420	A4d	Lu07	17858515	17860349	forward	1834	1473	490	7.92	−0.602	55.15	nu: 5, cp: 1, cy: 2, mt: 2, vac: 2, er: 1, gb:1
*LushsfA6a*	Lus10006618	A6a	Lu08	22068530	22070272	forward	1742	1185	394	5.56	−0.734	44.47	nu: 14
*LushsfA6b*	Lus10039376	A6b	Lu10	3252307	3253927	forward	1620	1122	373	5.11	−0.650	42.35	nu: 13, cp: 1
*LushsfA7a*	Lus10000492	A7a	Lu14	4577433	4578881	forward	1448	1125	374	5.52	−0.737	42.24	nu: 13
*LushsfA7b*	Lus10014698	A7b	Lu01	26411220	26412688	reverse	1468	1137	378	5.51	−0.780	42.96	nu: 14
*LushsfA7c*	Lus10022083	A7c	unmapped	-	-	reverse	1410	1131	376	5.75	−0.744	43.11	nu: 14
*LushsfA8a*	Lus10003707	A8a	Lu03	4843561	4845792	reverse	2231	1122	373	4.97	−0.703	42.42	nu: 13, per: 1
*LushsfA8b*	Lus10001591	A8b	Lu04	8648138	8650354	reverse	2216	1122	373	4.78	−0.724	42.72	nu: 13, per: 1
*LushsfA9a*	Lus10027627	A9a	Lu11	14143005	14144526	forward	1521	1422	473	5.03	−0.697	53.45	nu: 14
*LushsfA9b*	Lus10011941	A9b	Lu10	7901682	7903160	reverse	1478	1377	458	5.19	−0.696	51.76	nu: 14
*LushsfB1a*	Lus10019348	B1a	Lu02	1244140	1247030	forward	2890	273	822	4.81	−0.995	30.15	nu: 14
*LushsfB1c*	Lus10009351	B1c	Lu13	5055375	5058490	forward	3115	852	283	4.84	−0.991	31.44	nu: 11, cp: 1, cy: 1, pm: 1
*LushsfB2a*	Lus10014994	B2a	Lu14	19043189	19044240	forward	1051	924	307	5.58	−0.694	33.51	nu: 14
*LushsfB2b*	Lus10008007	B2b	Lu07	6384378	6385552	reverse	1174	1098	365	4.80	−0.554	39.28	nu: 13, per: 1
*LushsfB2d*	Lus10024508	B2d	Lu09	7452072	7454461	reverse	2389	1227	408	5.27	−0.499	44.34	nu: 13, per: 1
*LushsfB4a*	Lus10042646	B4a	Lu09	17698210	17699546	reverse	1336	1233	410	8.25	−0.656	45.07	nu: 14
*LushsfB4b*	Lus10036062	B4b	Lu01	503457	505257	reverse	1800	951	316	6.21	−0.899	36.54	nu: 14
*LushsfB4c*	Lus10001133	B4c	Lu10	13207020	13208344	reverse	1324	1215	404	7.84	−0.704	44.83	nu: 14
*LushsfB5a*	Lus10011185	B5a	Lu15	837963	839813	forward	1850	615	204	9.32	−0.812	23.78	nu: 13.5, cy_nu: 0.5
*LushsfB5b*	Lus10018488	B5b	Lu08	23772105	23774084	reverse	1979	603	200	9.27	−0.668	23.19	nu: 12.5, cy_nu: 7, cp 1
*LushsfC1a*	Lus10023636	C1a	Lu11	15833912	15834932	forward	1020	921	306	7.78	−0.715	34.63	nu: 14
*LushsfC1b*	Lus10034907	C1b	Lu12	8134272	8135285	forward	1013	924	307	6.98	−0.695	34.46	nu: 14

Chromosomal location estimated as per You *et al*.^10^.

pI: isoelectric point, GRAVY: Grand average of hydropathicity, Mw(kD): Molecular weight in kilo Dalton, Subcel loc and no. NN: Subcellular location and number of nearest neighbours, nu: nucleus, mt: mitochondria, cp: chloroplast, per: peroxisome, cy: cytosol, vac: vacuole, ER: endoplasmic reticulum, gb: golgi body, cy_nu: dual localization cytosol and nucleus, pm: plasma membrane.

### Chromosomal distribution and gene duplications

**Figure 1:**
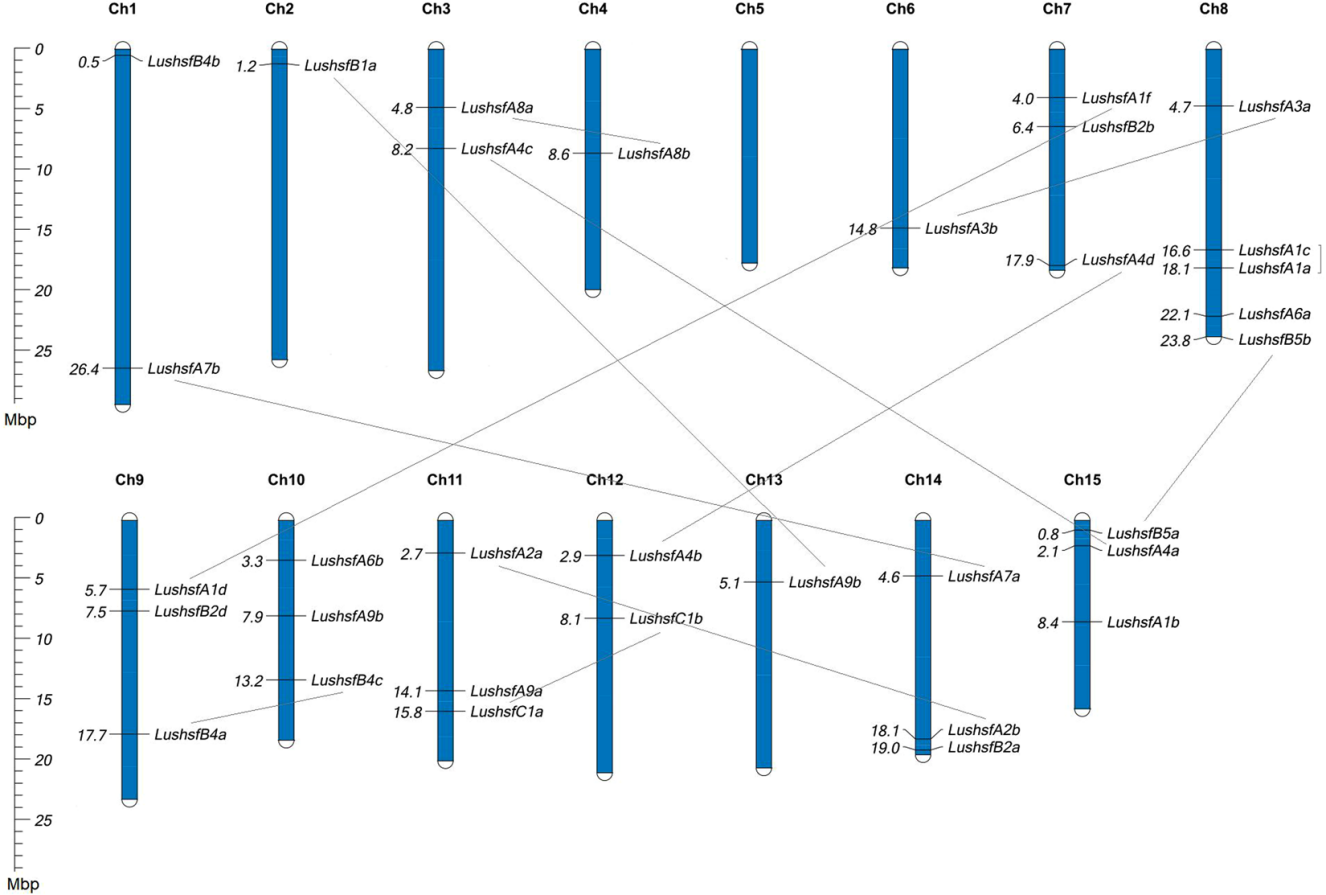
Chromosomal locations and duplication of *LusHSF* genes. Each bar represents the flax chromosome with the chromosome number shown above the bars. Chromosomal lengths are represented in Mbp. All 34 *LusHSF* genes are mapped on 14 out of the 15 flax chromosomes. The numbers on the left side of the chromosomes represent their physical positions in Mbp from top to bottom. Putative paralogous *LusHSF* genes are depicted through connected lines.

The genomic coordinates of the *LusHSF* genes on the scaffolds and flax chromosomes^10^ allowed us to estimate the physical location of these genes. Except for one gene, we found all *LusHSF* genes were randomly distributed on 14 out of the 15 flax chromosomes (Fig. 1). *LushsfA7c*, which is located on scaffold 87, remains unmapped because the entire scaffold has yet to be mapped on any chromosome. Not a single *LusHSF* gene was mapped on chromosome 5, while chromosome 8 consists of a maximum of five *LusHSF* genes. Four chromosomes, *viz*. 2, 4, 6 and 13, consisted of one *LusHSF* gene each. Gene expansion by duplication of the *LusHSFs* was checked through sequence homology analysis and their distribution patterns on the chromosomes. These analyses disclosed that twelve *LusHSF* genes have homologous gene pairs with >70% sequence identity and >90% query coverage. Eleven *LusHSF* genes have their duplicate counterparts (paralogues) distributed in separate chromosomes, while one pair, *viz*. *LushsfA1c* and *LushsfA1a*, are located on the same chromosome (Fig. 1). To further investigate whether this interspersed pattern of gene duplication resulted from segmental gene duplications, we compared *LusHSF* genes and their adjacent genomic regions using the GEvo tool of the CoGe database. Most of the putative *LusHSF* paralogues and their adjacent regions evolved because of local genomic rearrangements or microcolinearity (see Supplementary Fig. S1). This result indicates that segmental duplication played a significant part in the expansion of the *LusHSF* genes.

### Phylogenetic relationships of LusHSFs

**Figure 2:**
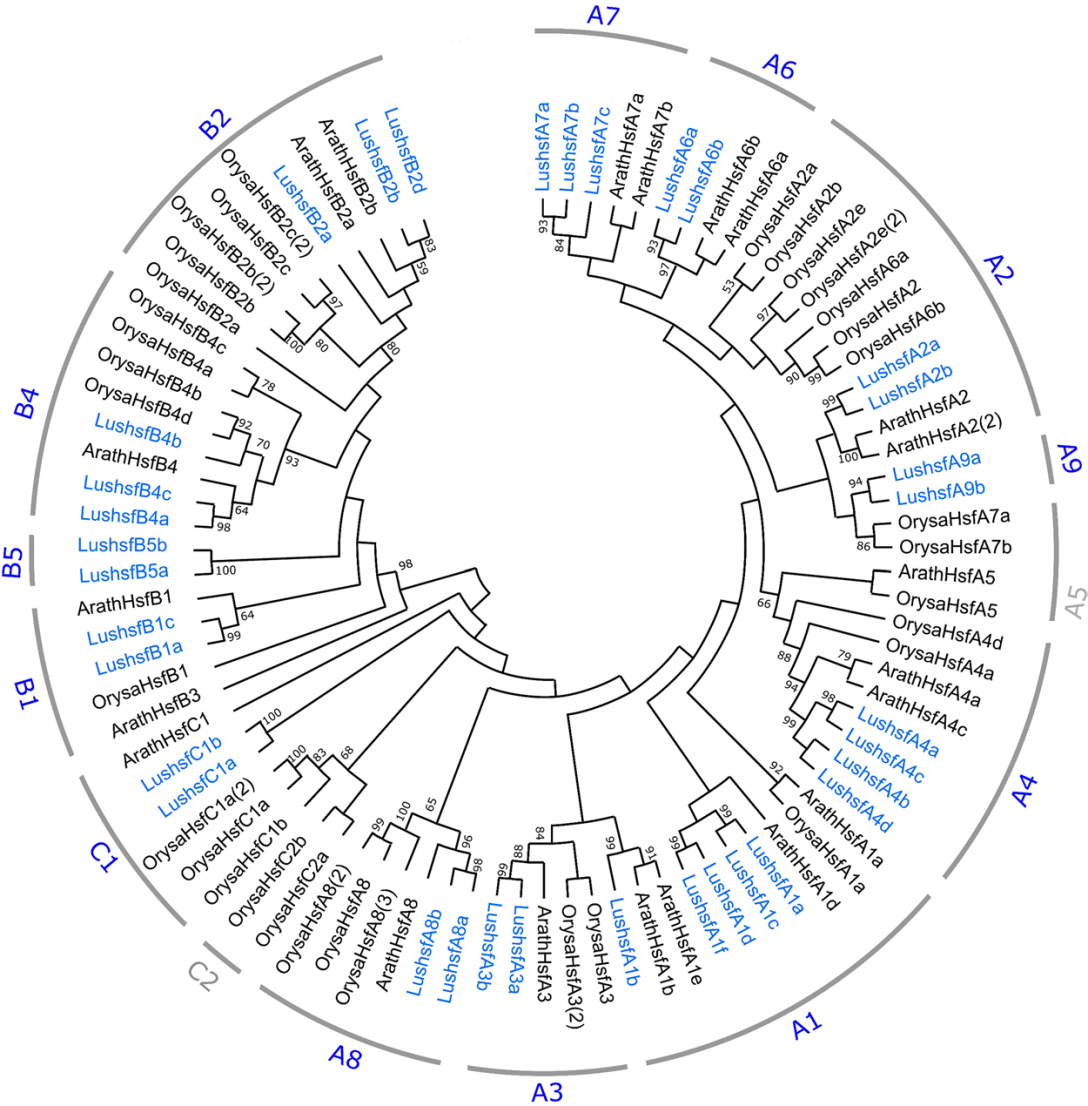
Phylogenetic clustering of LusHSFs, AtHSFs and OsHSFs. The phylogenetic relationship tree was inferred from the Maximum Likelihood (ML) method and JTT + G + I matrix-based model in MEGA-X. Domain-centric alignment of amino acid sequences from DBD and OD domains were performed using the MUSCLE algorithm with maximum 16 iterations. Thirty-four LusHSF, 21 AtHSF and 33 OsHSF proteins were clustered into 3 broad classes A, B, and C and 15 sub-classes within. Sub-groups marked in grey did not consist any LusHSFs. Bootstrap support values of >50% are shown on the nodes.

Employing multiple sequence alignment to *Arabidopsis thaliana* HSF (AtHSF) and *Oryza sativa* (OsHSF) proteins, the LusHSF proteins were classified into diverse groups, and a Maximum Likelihood (ML) tree was constructed based on highest log likelihood score (−4581.19) (Fig. 2). The best amino acid substitution model was found Jones-Taylor-Thornton (JTT) with lowest Bayesian Information Criterion (BIC) score of 14963.17. As per the phylogenetic tree, the LusHSFs were clustered into three broad groups, A, B, and C, and a total of 13 sub-groups, A1, A2, A3, A4, A6, A7, A8, A9, B1, B2, B4, B5 and C1, according to the HSF proteins grouped in clusters. These groupings were supported by high bootstrap values (>90%). All of the LusHSFs in the phylogenetic tree corroborated the classifications obtained from the HEATSTER database (Table 1). Neither of the LusHSF proteins was clustered in A5 and C2 sub-groups. Two LusHSF proteins, LushsfB5a and LushsfB5b, clustered separately in B5 sub-group, whereas AtHSFs and OsHSFs lack members from B5. Sub-group A1 comprised the most LusHSF proteins (five), followed by sub-group A4 (four). In the comparative phylogenetic analysis of HSFs with other plant species from the Malpighiales order and other fibre crops (such as cotton and jute), LusHSFs were grouped distinctly from the other proteins (see Supplementary Fig. S2).

### Organization of gene structure

**Figure 3:**
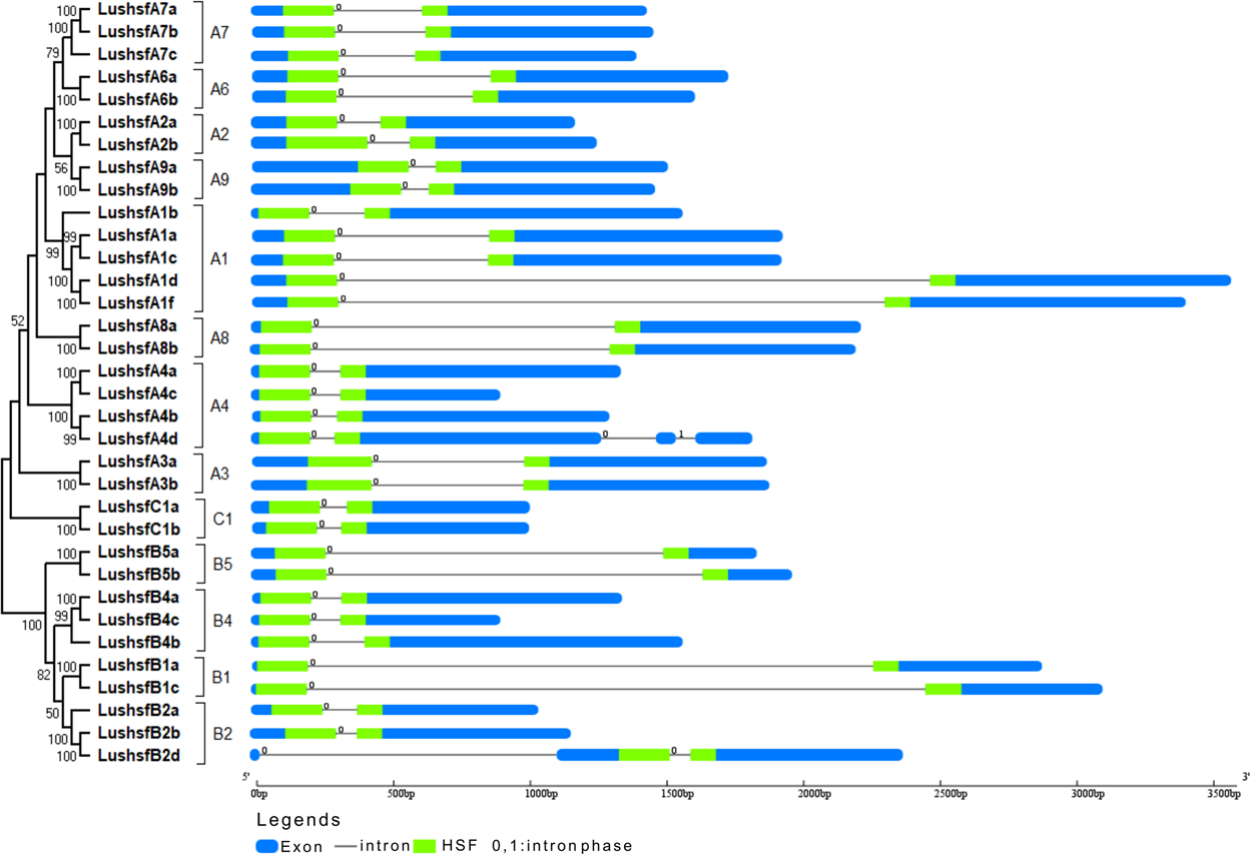
Gene structure showing the distribution of exons and introns of *LusHSF* genes. A phylogenetic ML tree rectangular diagram of *LusHSFs* genes is shown on the left. The lengths of the boxes and lines were scaled based on gene length. Blue bars represent exons, while thin black lines indicate introns. The green bars denote the position of the HSF DBD on exons. The numbers indicate splicing phases of *LusHSF* genes: 0 for phase 0 and 1 for phase 1.

The gene structure pattern, including the exon and introns on the *LusHSF* genes, was analysed by comparing the respective coding sequence and genomic sequences (Fig. 3). Introns were found in all 34 *LusHSF* genes, which ranged from one to three. The pattern of occurrence, position and length of the introns were found similar among the *LusHSFs* grouped under different sub-categories. The closely associated members of the same *HSF* group shared similar intron numbers and lengths, except for the *LushsfA4d* and *LushsfB2d*. A maximum of three introns was observed in the *LushsfA4d* sequence. The longest intron sequence (2.263 kbp) was found in *LushsfB1c*, followed by *LushsfA1d* (2.169 kbp). The smallest intron length, 76 bp, was observed in the *LushsfB2d* gene. In all *LusHSF* genes, an intron sequence was observed within the HSF-DBD, thus splitting the domain into two. The splicing phase class of all the introns within the HSF-DBD was observed as ‘0’ (*i*.*e*., between two codons resulting in unchanged frames or intact codon), except in *LushsfA4d*, where an intron splicing phase in one of the three introns was observed as ‘1’ (*i*.*e*., splitting codons between the first and second nucleotides). The presence of a single intron within the HSF-DBD region is one of the common features of plant *HSFs* that might have a possible role in mediating alternate splicing in genes that encode diverse protein products.

### Conserved protein domain and motif predictions

**Figure 4:**
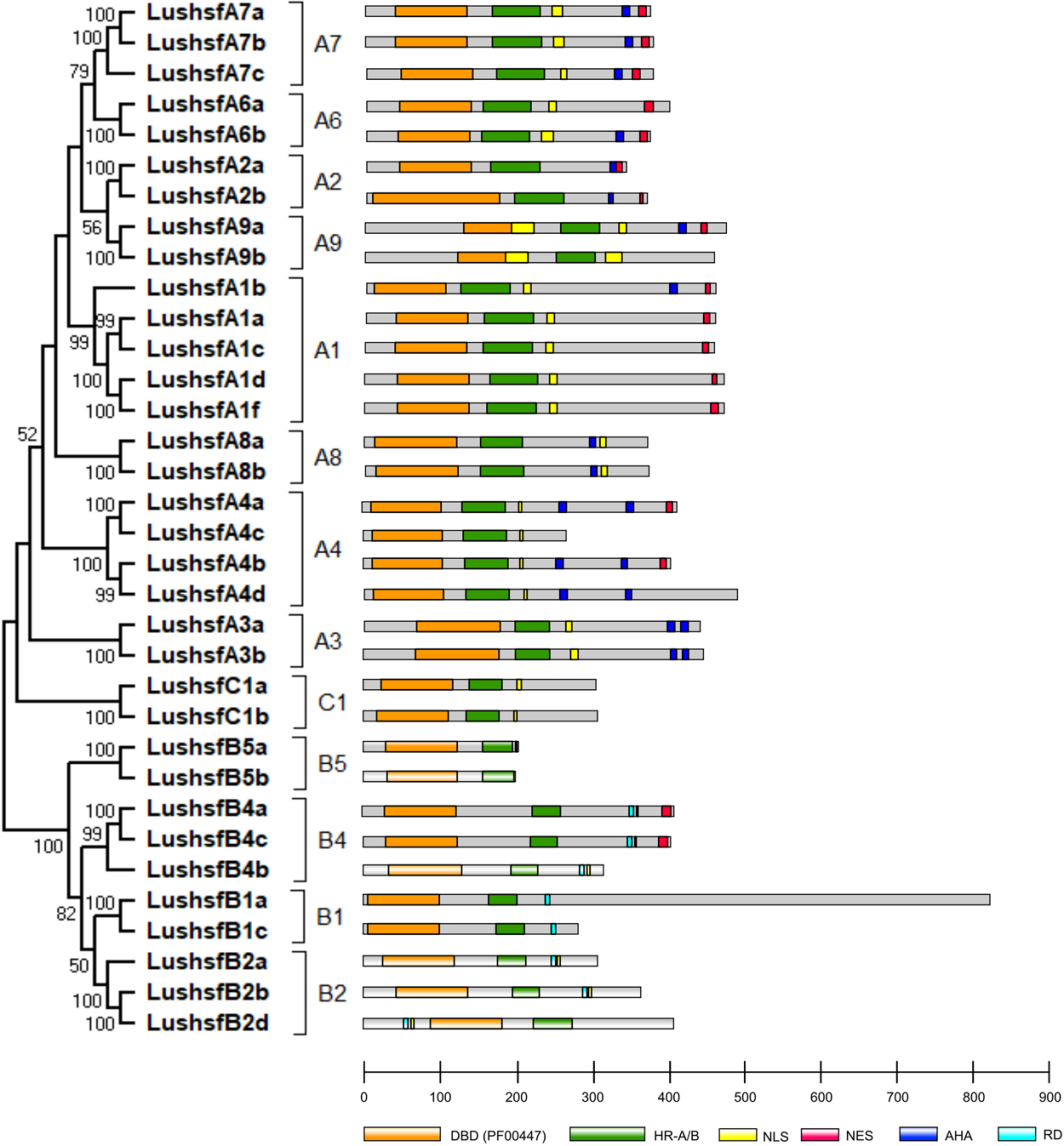
Distribution of conserved domains of the LusHSF proteins. A phylogenetic ML tree rectangular diagram of LusHSFs is shown on the left. Proteins with DBD and OD (HR-A/B motif) were scaled according to their lengths. Domain and motif legends are provided below the protein-length scale. For the detailed positions of the domains and motifs, see Table 2.

A systematic examination of all 34 LusHSF protein sequences revealed positions and sequences of discrete conserved motifs and domains (Table 2 and Fig. 4). Six types of conserved domains, DBD, OD (HR-A/B), NLS, NES, AHA, and RD, were identified within the LusHSF protein sequences. Except in one protein (LushsfB2d), the DBD was found at the N-terminus of all the LusHSF proteins, followed by the HR-A/B motif and other conserved motifs. In LushsfB2d, RD and NLS motifs precede the DBD. This finding indicated that the DBD and OD, comprising the HR-A/B motif, are the highly conserved domains on LusHSF proteins, followed by the NLS and NES domains. The NLS and NES domains, which are responsible for translocating HSF proteins to the nucleus, were found on most of the LusHSFs either individually or together, except in three proteins, LushsfB1c, LushsfB5b, and LushsfB1a (Table 2). A thorough look at the multiple sequence alignments of the DBD revealed a highly structured domain of conserved motifs that forms three bundles of alpha helices (*α*1, *α*2 and *α*3) and four antiparallel beta strands (see Supplementary Fig. S3a). However, minor differences in the DBD length and amino acid sequence insertions were observed in the DBD alignment, notably in the LushsfA2b sequence. Compared to the amino acid sequence alignment of the DBD, the HR-A/B motif, which forms a coiled-coil structure, was found to be more variable (see Supplementary Fig. S3b). Typically, HR-A and HR-B are two conserved motifs with sequence inserts between them. The HR-A motif was absent or partial in six of the 34 LusHSFs, while ten LusHSFs consisted of partial or no HR-B motifs.

**Table 2:** Conserved domains and motifs on LusHSF proteins.

LusHSF ID	Group	DBD	HR-A/B	NLS	NES	AHA1	AHA2	AHA3	AHA4	RD	NLS and NES domain sequences
LushsfA1a	A1a	39–134	141–233	236–253	436–450	—	—	—	—	—	NLS-SRRISESSKKRRLKQDGV; NES-PMENMDQLTQQMGLL
LushsfA1b	A1b	9–104	110–202	205–222	437–451	—	392–410	—	—	—	NLS-SRRIVGGTKKRRLPAHEG; NES-KVEGMNYLTEQMGLL
LushsfA1c	A1c	39–134	141–233	236–253	436–450	—	—	—	—	—	NLS-TRRISESSKKRRLKQDGV; NES-PMENMDQLTQQMGLL
LushsfA1d	A1d	43–138	147–239	242–259	449–463	—	—	—	—	—	NLS-NRRITETNKKRRLKQDRT; NES-KTEHVDQLTEQMGHL
LushsfA1f	A1f	43–138	147–239	242–259	449–463	—	—	—	—	—	NLS-NRRIAETNKKRRLKQDGT; NES-KSEHVDQLTEQMGHL
LushsfA2a	A2a	36–142	158–237	—	326–334	—	298–325	—	—	—	NES-QDLVDQMGFL
LushsfA2b	A2b	36–179	189–268	—	359–364	316–328	—	—	—	—	NES-QMGFL
LushsfA3a	A3a	68–186	193–260	264–273	—	—	—	—	—	—	NLS-TTRKKFIRHN
LushsfA3b	A3b	68–187	195–262	273–282	—	—	—	403–412	420–426	—	NLS-RTRKKFIRHN
LushsfA4a	A4a	6–118	123–202	206–225	396–410	336–372	376–393	—	—	—	NLS-RKRRLPRISCYDDPMLEDST; NES-NVNSLAGHIGPLTPA
LushsfA4b	A4b	6–118	126–205	206–225	386–400	327–363	368–383	—	—	—	NLS-RKRRLPRLGCIDDDPMKEDT; NES-SVNGLAGQVGPLTPA
LushsfA4c	A4c	6–118	123–202	206–225	—	—	—	—	—	—	NLS-RKRRLPRISCYDDPMLEDST
LushsfA4d	A4d	6–119	126–205	210–229	—	331–367	372–387	—	—	—	NLS-RKRRLPRLDCIDDDPMKEDT
LushsfA6a	A6a	38–139	144–225	227–249	362–380	—	—	—	—	—	NLS-HQKGIMRELENAITKKRRRRPID; NES-RNVDVLLEQLGFFLPPPY
LushsfA6b	A6b	36–137	142–223	225–237	356–373	325–338	—	—	—	—	NLS-HQKGIMRELENAITKKRRRRPID; NES-GNVDVLVEQLGFLDSDIM
LushsfA7a	A7a	34–135	158–239	241–251	356–373	335–247	—	—	—	—	NLS-QRKEKRKELEEALSKKRRRPID; NES-EDVNTLAEQLGYLSSSSP
LushsfA7b	A7b	34–135	159–240	242–263	360–377	339–351	—	—	—	—	NLS-QRKEKRKELEEALSKKRRRPIE; NES-EDVNTLAEQLGYLSSSSP
LushsfA7c	A7c	40–141	162–243	255–265	347–364	323–335	—	—	—	—	NLS-NNKRRRRRPIG; NES-EAVTALAEQLGYLPIRLK
LushsfA8a	A8a	10–110	137–208	209–216	—	—	—	—	—	—	NLS-SWRMAEPG
LushsfA8b	A8b	10–109	137–208	209–216	—	—	—	—	—	—	NLS-SWRMAEPG
LushsfA9a	A9a	128–231	249–342	—	—	403–431	436–456	—	—	—	—
LushsfA9b	A9b	120–223	243–336	—	—	—	—	—	—	—	—
LushsfB1a	B1a	1–100	165–213	—	—	—	—	—	—	236–244	—
LushsfB1c	B1c	1–100	173–222	273–283	245–253	—	—	—	—	247–253	NLS-GPRAKEIKICY
LushsfB2a	B2a	19–136	167–216	255–264	—	—	—	—	—	237–254	NLS-KKRGREEGGG
LushsfB2b	B2b	37–154	186–235	296–305	——	—	—	—	—	279–295	NLS-KRARIEEEEE
LushsfB2d	B2d	82–199	229–278	339–348	—	—	—	—	—	321–338	NLS-KRVRIDEEEE
LushsfB4a	B4a	27–126	221–267	357–363	—	—	—	—	—	341–356	NLS-NNHNHN
LushsfB4b	B4b	30–133	191–237	293–297	—	—	—	—	—	275–292	NLS-KKRQL
LushsfB4c	B4c	27–126	216–262	356–359	381–394	—	—	—	—	337–355	NLS-HHHN; NES-SSKSHRLVLEKDDL
LushsfB5a	B5a	25–136	156–196	199–204	—	—	—	—	—	—	NLS-KNRRTC
LushsfB5b	B5b	26–137	157–197	—	—	—	—	—	—	—	—
LushsfC1a	C1a	20–117	134–197	198–212	—	—	—	—	—	—	NLS-KLDHRKKRCLMTSIS
LushsfC1b	C1b	15–113	129–192	193–207	—	—	—	—	—	—	NLS-KLDHRKKRCLMALVS

DBD: DNA-bind domain; HR-A/B: heptad repeat A (N-terminus) or B (C-terminus) domain; NLS: nuclear localization signal; NES: nuclear export signal; AHA: aromatic and hydrophobic amino acid residues embedded in an acidic context; RD: repressor domain.

### DNA interaction interface predictions on LusHSF proteins

Identification of protein-protein interaction sites and protein-DNA binding sites on the LusHSF proteins through the PredictProtein server showed a change in the number and location of the active sites (see Supplementary Fig. S4a). Except in LushsfA3a, all LusHSF amino acid sequences were predicted to have these macro-molecular interaction sites. Twenty-two out of the 34 LusHSFs consisted of polynucleotide binding sites. The diversity of the DNA contact points or active DNA binding sites on the LusHSF proteins was further analysed utilizing the protein model-based server TFmodeller, which revealed that most of the LusHSF proteins have DNA contact sites in the N-ring, *i*.*e*., purine or pyrimidine through six amino acid interface residues (Table 3). These DNA contact sites were predicted from a matrix of homologous interface contacts by comparing structurally related protein-DNA complexes. The six amino acid residues included serine (S), glutamine (Q), asparagine (N), threonine (T) and two arginine (R) residues. These residues are conserved in all the contact sites, except for LushsfA8b and LushsfA8a, where threonine (T) is replaced with isoleucine (I). The only notable diversity of these DNA contact sites is generally owing to the positional variance of these six amino acid residues in the protein sequence, typically residing between 60 and 221 amino acids from the N-terminus. With our findings, the template human heat shock factor protein model 5d5v_B chain was compared to reveal protein-DNA interface sites on most of the LusHSFs (see Supplementary Fig. S4b). Four LusHSF proteins found no homologous templates to model the protein-DNA interface. The specificity, which represents the evolutionary proportion of sequence-specific contacts for the complex, was almost comparable, 0.26–0.27 (except in four non-homologous LusHSFs, 0.04), in all the LusHSFs, but the level of entropy varied from 0.73 to 1.00.

**Table 3:** Details of DNA binding site predictions on LusHSF proteins.

HSF_ID	No of contact sites	No of contact sites in N-ring	N-ring contact sites (position and amino acid)	Specificity	Entropy	PDB_ID of model
LushsfA1a	22	6	93-S, 96-R, 97-Q, 99-N, 100-T, 131-R	0.27	0.73	5d5v_B
LushsfA1b	22	6	63-S, 66-R, 67-Q, 69-N, 70-T, 101-R	0.27	0.87	5d5v_B
LushsfA1c	22	6	93-S, 96-R, 97-Q, 99-N, 100-T, 131-R	0.27	0.81	5d5v_B
LushsfA1d	22	6	97-S, 100-R, 101-Q, 103-N, 104-T, 135-R	0.27	0.83	5d5v_B
LushsfA1f	22	6	97-S, 100-R, 101-Q, 103-N, 104-T, 135-R	0.27	0.86	5d5v_B
LushsfA2a	22	6	97-S, 100-R, 101-Q, 103-N, 104-T, 135-R	0.27	0.86	5d5v_B
LushsfA2b	24	1	172-R	0.04	0.88	—
LushsfA3a	24	1	176-R	0.04	0.91	—
LushsfA3b	24	1	177-R	0.04	0.82	—
LushsfA4a	22	6	64-S, 67-R, 68-Q, 70-N, 71-T, 102-R	0.27	0.86	5d5v_B
LushsfA4b	22	6	64-S, 67-R, 68-Q, 70-N, 71-T, 102-R	0.27	0.94	5d5v_B
LushsfA4c	22	6	64-S, 67-R, 68-Q, 70-N, 71-T, 102-R	0.27	0.89	5d5v_B
LushsfA4d	22	6	64-S, 67-R, 68-Q, 70-N, 71-T, 102-R	0.27	0.84	5d5v_B
LushsfA6a	23	6	97-S, 100-R, 101-Q, 103-N, 104-T, 135-R	0.26	0.82	5d5v_B
LushsfA6b	23	6	95-S, 98-R, 99-Q, 101-N, 102-T, 133-R	0.26	0.89	5d5v_B
LushsfA7a	22	6	93-S, 96-R, 97-Q, 99-N, 100-T, 131-R	0.27	0.83	5d5v_B
LushsfA7b	22	6	93-S, 96-R, 97-Q, 99-N, 100-T, 131-R	0.27	0.87	5d5v_B
LushsfA7c	22	6	99-S, 102-R, 103-Q, 105-N, 106-T, 137-R	0.27	0.86	5d5v_B
LushsfA8a	22	6	66-S, 69-R, 70-Q, 72-N, 73-I, 104-R	0.27	0.87	5d5v_B
LushsfA8b	22	6	66-S, 69-R, 70-Q, 72-N, 73-I, 104-R	0.27	0.97	5d5v_B
LushsfA9a	22	6	183-S, 186-R, 187-Q, 189-N, 190-T, 221-R	0.27	0.89	5d5v_B
LushsfA9b	22	6	175-S, 178-R, 179-Q, 181-N, 182-T, 213-R	0.27	0.90	5d5v_B
LushsfB1a	23	6	60-S, 63-R, 64-Q, 66-N, 67-T, 98-R	0.26	0.79	5d5v_B
LushsfB1c	24	1	111-R	0.04	0.93	-
LushsfB2a	22	6	79-S, 82-R, 83-Q, 85-N, 86-T, 117-R	0.27	0.88	5d5v_B
LushsfB2b	22	6	97-S, 100-R, 101-Q, 103-N, 104-T, 135-R	0.27	0.97	5d5v_B
LushsfB2d	22	6	142-S, 145-R, 146-Q, 148-N, 149-T, 180-R	0.27	0.97	5d5v_B
LushsfB4a	23	6	83-S, 86-R, 87-Q, 89-N, 90-T, 121-R	0.26	0.98	5d5v_B
LushsfB4b	22	6	90-S, 93-R, 94-Q, 96-N, 97-T, 128-R	0.27	0.97	5d5v_B
LushsfB4c	23	6	83-S, 86-R, 87-Q, 89-N, 90-T, 121-R	0.26	0.95	5d5v_B
LushsfB5a	23	6	83-S, 86-R, 87-Q, 89-N, 90-T, 121-R	0.26	1.00	5d5v_B
LushsfB5b	23	6	84-S, 87-R, 88-Q, 90-N, 91-T, 122-R	0.26	0.99	5d5v_B
LushsfC1a	23	6	76-S, 79-R, 80-Q, 82-N, 83-T, 114-R	0.26	0.89	5d5v_B
LushsfC1b	23	6	71-S, 74-R, 75-Q, 77-N, 78-T, 109-R	0.27	0.96	5d5v_B

S-serine; R-arginine, Q-glutamine, N-asparagine, T-threonine, I-isoleusine ‘–’ no model.

### Orthologues of *LusHSFs*, syntenic relationships and divergence time

**Figure 5:**
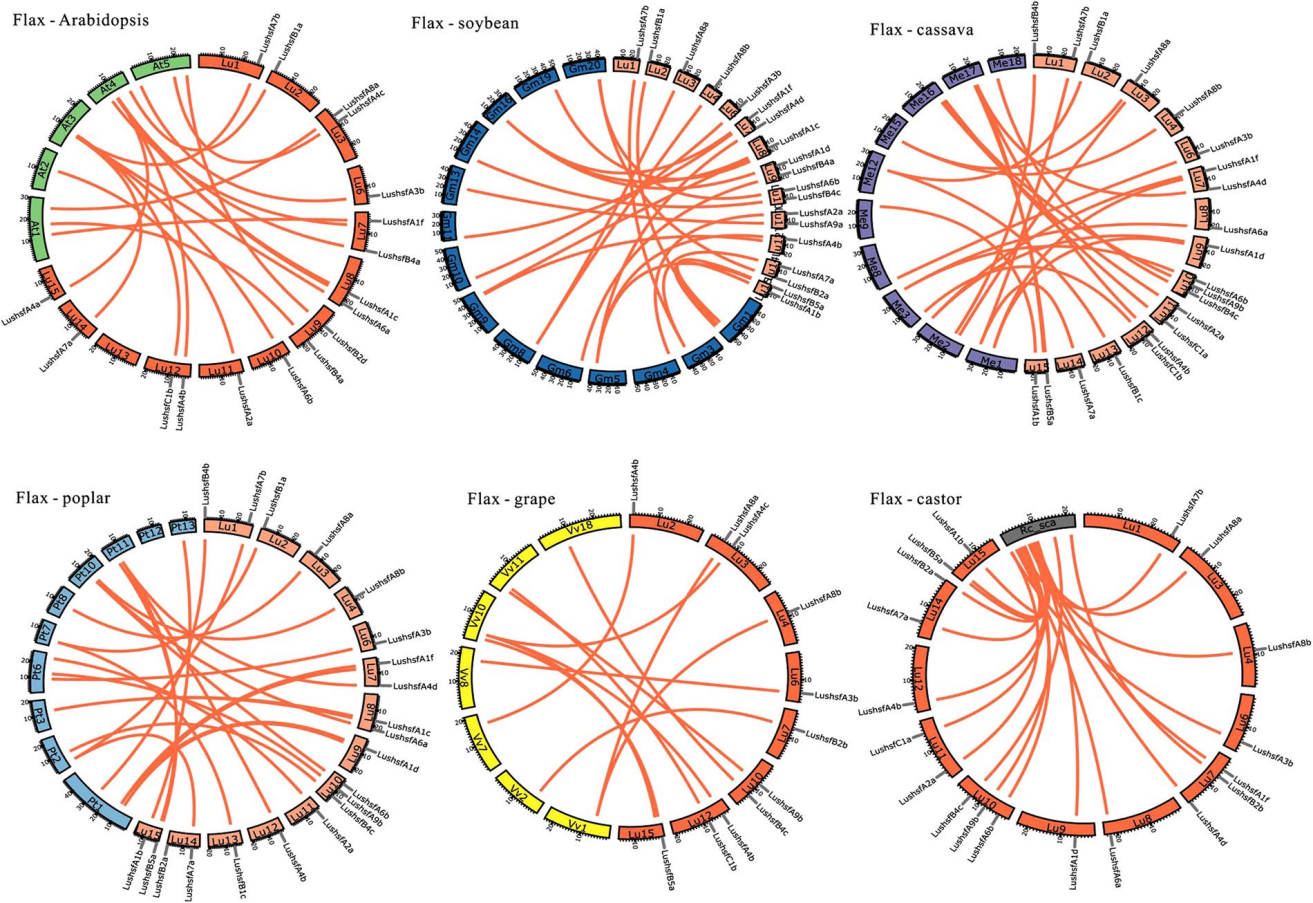
Syntenic relationships among putative orthologues and *LusHSF* genes. The syntenic relations of *LusHSF* genes to Arabidopsis, soybean, cassava, poplar, grape and castor were plotted using CIRCOS v0.69-5. The chromosomal positions of the syntenic *HSF* gene pairs are represented with red links. *LusHSF* genes are labeled on flax chromosomes, and the chromosome numbers are mentioned in the karyotype chords.

**Figure 6:**
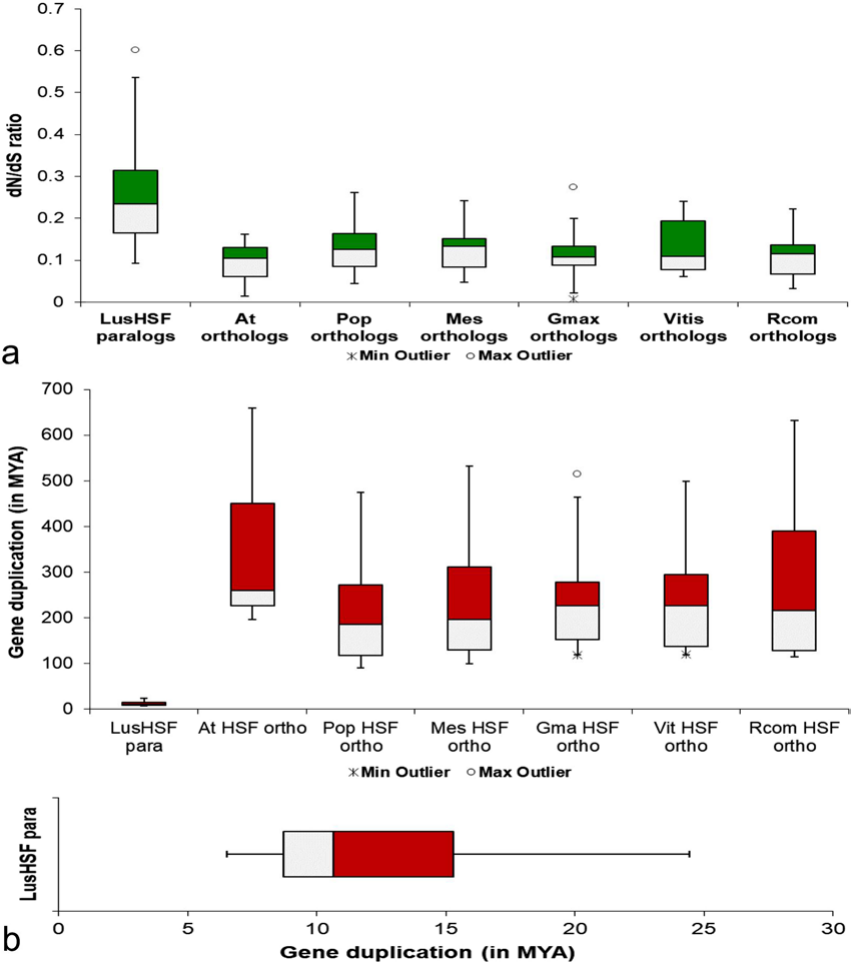
Box and whisker plots showing comparative distribution of (**a**) substitution rates of non-synonymous over synonymous site (d_*N*_/d_*S*_) and (**b**) estimated time of gene duplication (MYA) in putative paralogues and orthologues of *LusHSFs*. The top of the box or coloured region represents the 3rd quartile (Q3, maximum values) while the bottom of the box or white region represents the 1st quartile (Q1, lower values). The ends of whiskers represent maximum and minimum values 1.5 times above or lower the Q3 and Q1, respectively. The maximum and minimum outlier values are represented as open circles and star symbols, respectively. In (**b**), the detailed distribution of gene duplication times (in MYA) of putative *LusHSF* paralogous gene pairs are shown separately below the comparative figure.

Putative orthologues of *LusHSFs* genes were predicted using the reciprocal protein blast approach through crb-blast and OrthoFinder software. HSF proteins from three related plant systems, such as *Populus trichocarpa*, *Ricinus communis*, *Manihot esculenta*, and three additional plant systems where the *HSF* genes are well characterized, such as *A*. *thaliana*, *Vitis vinifera*, and *Glycine max*, were compared to LusHSF proteins. The crb-blast showed that 31 LusHSF proteins matched to 87 unique HSF hits. OrthoFinder placed the 34 LusHSF proteins into eleven orthogroups and matched to 140 HSF hits. Of the 34 LusHSF proteins, thirty-one (91.2%) were consistent in both programmes and had orthologues in at least one of these six species. A maximum of 17 LusHSF orthologues was related to both *P*. *trichocarpa* HSFs (36.2%) and *M*. *esculenta* HSFs (43.6%), while a minimum of nine orthologues (47.4%) was related to the *V*. *vinifera* HSFs (Supplementary Table S2. The synteny map of the above *LusHSF* orthologous genes revealed that these genes are conserved and are randomly assigned in most of the chromosomes of the orthologous species (Fig. 5). To determine the evolutionary status of the putative *LusHSF* gene paralogues and orthologues, the ratio of substitution rates of non-synonymous (d_*N*_) versus synonymous (d_*S*_) sites was computed for each pair of duplicated genes. The d_*N*_/d_*S*_ ratios computed for all the putative paralogues and orthologues varied from 0.0065 (*LushsfA3a*-*Glyma*.*09G190600*.*1*) to 0.6022 (*LushsfA3b*-*LushsfA3a*). The overall distribution of the d_*N*_/d_*S*_ ratios is presented in Fig. 6a and Supplementary Table S2. The average and median d_*N*_/d_*S*_ ratios were lowest, 0.096 and 0.105, for the putative *LusHSFs* and *Arabidopsis HSF* orthologues, respectively, while these values were highest, 0.268 and 0.234, for the putative *LusHSF* paralogues, respectively (Supplementary Table S2). In general, the d_*N*_/d_*S*_ ratio was <1.0, indicating that these duplicated genes are under negative or purifying selection pressure. The d_*N*_/d_*S*_ ratios were further used to predict gene duplication times in terms of million years ago (MYA) for each of these putative paralogous and orthologous gene pairs (Supplementary Table S2). The time for the gene duplication of *LusHSFs* (average ~12.5 MYA, median ~10.6 MYA) was observed as a more recent event than that for the divergence of the orthologues (Fig. 6b). The latest duplication time was estimated at ~6.5 MYA (*LushsfA7a*-*LushsfA7b*) and with oldest duplication time occurring ~24.5 MYA (*LushsfC1a*-*LushsfC1b*). The median values for the divergence of *LusHSFs* from the orthologues of *P*. *trichocarpa HSFs* were predicted as the latest (~186.2 MYA), while the earliest divergence time prediction was for orthologues from *Arabidopsis HSFs* (~259.7 MYA). Among the *LusHSF* orthologues analysed, five gene pairs, viz. *LushsfB1a*- *AT4G36990*.*1*, *LushsfA1a*- *Glyma*.*09G206600*.*2*, *LushsfC1b*- *Glyma*.*09G190600*.*1*, *LushsfA2a*-*29912*.*m005526*, and *LushsfB2a*-*30147*.*m014282*, showed d_*S*_ values > 10 and predicted highly conserved evolutionary times, dating back >1000 MYA.

### *Cis*-acting element localization on *LusHSF* promoters

Since the promoter of a gene often consists of *cis*-acting regulatory elements that confer its functional specificity, we analysed the distribution of *cis*-elements in the 1000 bp upstream promoter sequence of *LusHSF* genes. First, our analysis with the TSSP program Softberry showed that four out of 34 *LusHSF* promoters comprised unverified bases, thus restricting their lengths to less than 1000 bp for the analysis. Putative promoter positions based on the transcription start site (TSS) were predicted in a total of 24 (70.6%) *LusHSF* upstream sequences (Table 4). Four of these sequences showed more than one putative TSS position. The location of the putative TATA box sequences in the 14–38 bp region upstream of the TSS was predicted in 23 out of 24 *LusHSF* promoters. Enhancer elements were predicted in 14 of the *LusHSF* promoters, of which two promoters consisted of more than one enhancer element. Next, our analysis of the distribution of *cis*-acting regulatory elements in the promoter sequences of *LusHSFs* demonstrated the existence of various regulatory elements related to the abiotic-stress response (Table 4). These elements include *ABRE* (*abscisic acid responsive element*), *CCAAT-box*, *DRE/CRT/CBF* (*dehydration-responsive element/C-repeat/C-repeat binding factors*), *HSE*, *LTRE* (*low-temperature response element*), *MBS* (*MYB-binding site*), and *PRECONSCRHSP70* (*plastid response element in the promoters of heat shock protein 70* *A*). Although the software programs PlantCARE and PLACE both predicted abiotic stress-related regulatory elements, in a majority of the *LusHSF* promoters, both programs varied in the number of predicted elements. PlantCARE predicted a smaller number of elements compared to PLACE. In agreement with the PlantCARE program, a considerable number of *LusHSF* promoters were found to consist of *HSE* and *LTRE*, which are linked to impart tolerance to high and low temperatures. In addition, a significant number of elements of *ABRE*, *MBS*, and *TC-rich repeats* were also located, which are likely to be induced under dehydration stress. Each of two *LusHSF* promoters consisted of *DRE* and *CCAAT box* elements; the former is responsible for dehydration stress tolerance, and the latter is involved in interactions with an *HSE* element to enhance heat shock promoter activity. The program PLACE predicted a considerable number of MYB/MYC transcription factor-binding sites ranging from zero to 31, followed by *CRT/DRE/CBF* and *LTRE*. A significant number of *cis*-elements associated with *heat shock protein 70* (*HSP70*) were also located on the *LusHSF* promoters by the program PLACE. Altogether, the above results show that the *LusHSF* promoters are enriched with numerous potential *cis*-acting elements related to the abiotic-stress response.

**Table 4:** Details of promoter analysis in the 1000 bp upstream sequence of the LusHSF genes. Position of first nucleotide of putative TSS/TATA box/Enhancer from the 5’ end of the upstream sequence analyzed and not from the start codon. NP- No prediction; PRECON70#- PRECONSCRHSP70.

Gene_ID	Prom_len	TSSP (SOFTBERRY) predictions*			Number of cis-elements (PlantCARE)							Number of cis-elements (PLACE				
		TSS	TATA box	Enhancer	ABRE	CCAAT-box	DRE	HSE	LTR	MBS	TC-rich repeats	ABRE	MYB	LTRE	PRECON70#	CRT/DRE/CBF
LushsfA1a	1000	NP	—	—	2	0	0	0	0	0	1	2	13	1	1	3
LushsfA1b	1000	749	725	—	1	0	0	0	2	0	0	7	14	5	1	5
LushsfA1c	1000	550	512	—	2	0	0	1	2	0	1	1	14	2	0	0
LushsfA1d	1000	931	916	—	0	0	0	1	0	2	0	2	15	2	1	2
LushsfA1f	1000	450	415	—	0	0	0	2	0	1	1	1	21	4	2	2
LushsfA2a	1000	892	855	761	1	0	0	0	0	3	0	0	18	0	0	2
LushsfA2b	1000	NP	—	—	0	0	0	0	0	4	0	0	18	3	2	3
LushsfA3a	1000	940	927	869	2	0	0	0	0	2	1	3	24	3	2	6
LushsfA3b	1000	941	927	869	1	0	0	0	1	0	0	3	24	2	1	2
LushsfA4a	1000	NP	—	—	0	1	0	0	0	0	1	0	15	0	2	0
LushsfA4b	1000	NP	—	—	0	0	0	0	0	1	1	0	15	1	2	6
LushsfA4c	1000	NP	—	—	0	0	0	0	0	0	2	0	16	0	1	1
LushsfA4d	1000	292	256	—	0	0	0	1	0	1	2	0	18	1	3	6
LushsfA6a	1000	895; 461	860; 425	928	0	0	0	0	0	2	2	1	11	0	3	8
LushsfA6b	1000	905	868	926	0	0	0	0	0	1	1	0	17	1	2	7
LushsfA7a	1000	828	795	155; 874	0	0	0	1	1	0	0	0	3	1	0	0
LushsfA7b	1000	822	789	861	0	0	0	0	1	1	2	0	19	2	0	4
LushsfA7c	748	438	412	696	0	0	0	0	0	1	1	2	11	0	1	1
LushsfA8a	1000	NP	—	—	0	0	0	1	2	1	0	4	12	3	2	3
LushsfA8b	1000	NP	—	—	0	0	0	2	0	6	0	2	27	2	2	11
LushsfA9a	1000	633	616	—	4	0	0	6	1	1	1	4	18	2	3	2
LushsfA9b	939	NP	—	—	4	0	0	0	0	1	0	4	25	1	2	2
LushsfB1a	1000	607; 192	575; 160	615	0	0	0	0	0	0	0	1	14	1	1	0
LushsfB1c	1000	626; 175	592; 156	632	0	0	0	2	0	1	1	0	9	1	4	0
LushsfB2a	1000	NP	—	—	0	0	0	0	1	1	0	1	9	4	0	3
LushsfB2b	1000	578	540	582	2	0	0	1	1	0	2	3	12	2	0	0
LushsfB2d	1000	425	395	231	0	0	0	0	0	1	1	2	7	0	2	0
LushsfB4a	1000	223	203	922; 315	0	0	0	2	2	0	2	0	11	3	0	0
LushsfB4b	1000	NP	—	—	0	0	1	0	1	3	0	0	31	3	1	5
LushsfB4c	287	209	—	—	0	0	0	1	1	0	0	0	0	1	0	0
LushsfB5a	769	723	697	—	1	0	0	2	0	0	0	2	3	0	0	1
LushsfB5b	1000	945	931	—	1	0	0	0	0	1	0	2	24	1	1	3
LushsfC1a	1000	948	913	—	2	0	0	1	1	1	0	6	10	4	1	1
LushsfC1b	1000	925; 108	892; 70	936	2	0	0	0	1	3	1	3	17	2	1	4

### Gene expression dynamics of *LusHSFs* in different tissues

**Figure 7:**
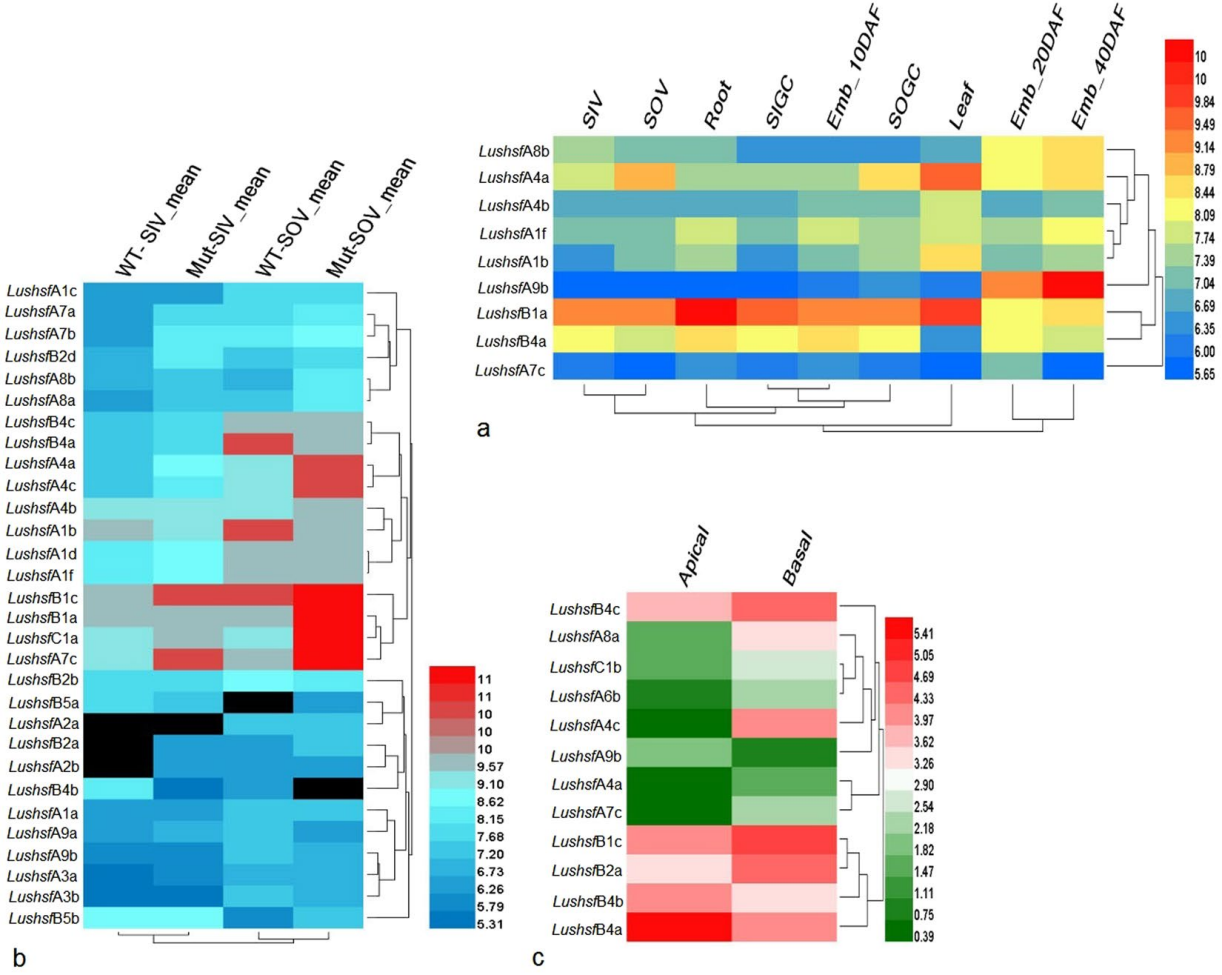
Heat map and hierarchical clustering of digital gene expression of *LusHSF* genes in different flax tissues. (**a**) *LusHSF* corresponding gene IDs were derived from the microarray data under GEO accession no. GSE21868. The mean of RMA-normalized, averaged gene-level signal intensity (log_2_) values were plotted using the Heatmap Illustrator (HemI v.1.0). Tissue includes SIV: stem inner tissue from the vegetative stage; SOV: stem outer tissue from the vegetative stage; root; leaf; SIGC: inner stem at the green capsule stage; SOGC: outer stem at the green capsule stage; and embryo at 10, 20, and 40 days post flowering. (**b**) The normalized signal intensity values of the *LusHSF* genes derived from the transcriptome data under GEO accession no. GSE61311 is plotted as a heat map. Digital samples include WT-SIV: inner stem tissue from the vegetative stage of wild-type plants; mut-SIV: inner stem tissue from the vegetative stage of lignified bast fibre mutant plants; WT-SOV: outer stem tissue from the vegetative stage of wild type plant; and mut-SOV: outer stem tissue from the vegetative stage of lignified bast fibre mutant plant. (**c**) Heat map generated for the *LusHSFs* derived from RNA-seq data (Accession no. GSE80718) using the log_2_ transformed average FPKM values. In all heat map plots, the coloured bars shown on the right represent their expression levels.

A homology search of *LusHSF* genes against the microarray data (Accession no. GSE21868) revealed only nine high-quality unigene hits (>95% identity). The fewer number of *LusHSF* hits to the microarray data could be attributed to the expressed sequence tags (ESTs) of the Hermes cultivar used to develop the array rather than the flax genome of CDC Bethune. Nonetheless, these nine *LusHSF* genes revealed a differential gene expression pattern in different flax tissues (Fig. 7a). On a closer look, the *LushsfB1a*, which belongs to the B1 group, was found to have higher gene expression in most of these tissues, while *LushsfA7c* was expressed at low levels. *HSF* genes from the B1 group are heat inducible and are known for their role in repressing other *HSF* genes under non-heat conditions. Interestingly, the *LushsfA9b* gene, which belongs to the A9 group, was less abundant in all tissues but was highly expressed in the late embryo developmental stages. *HSF* genes from A9 groups are known for their involvement in seed development. Similarly, in another microarray dataset (GSE61311), eight *LusHSFs* exhibited differential expression patterns in inner and outer stem tissues at the vegetative stage of the wild and mutant genotypes (Fig. 7b). Compared to the microarray data of *LusHSFs*, the differentially expressed transcriptome resources from the shoot apex of the flax variety CDC Bethune (GSE80718) showed higher hits of 27 *LusHSF* genes. Twelve of these *LusHSF* genes showed differential expression patterns in the apical and basal tissues (Fig. 7c). Five *LusHSF* genes were expressed in abundance and three genes showed low expression in both tissues. However, four *LusHSF* genes showed contrasting expression patterns in these tissues. The hierarchical clustering of the *LusHSF* genes in all the above digital gene expression analyses was found in accordance with their expression patterns. From the above digital gene expression analysis, we speculate that the majority of *LusHSF* genes differ in their expression patterns in various flax tissues and growth stages.

### Expression pattern of *LusHSF* genes under HT stress

**Figure 8:**
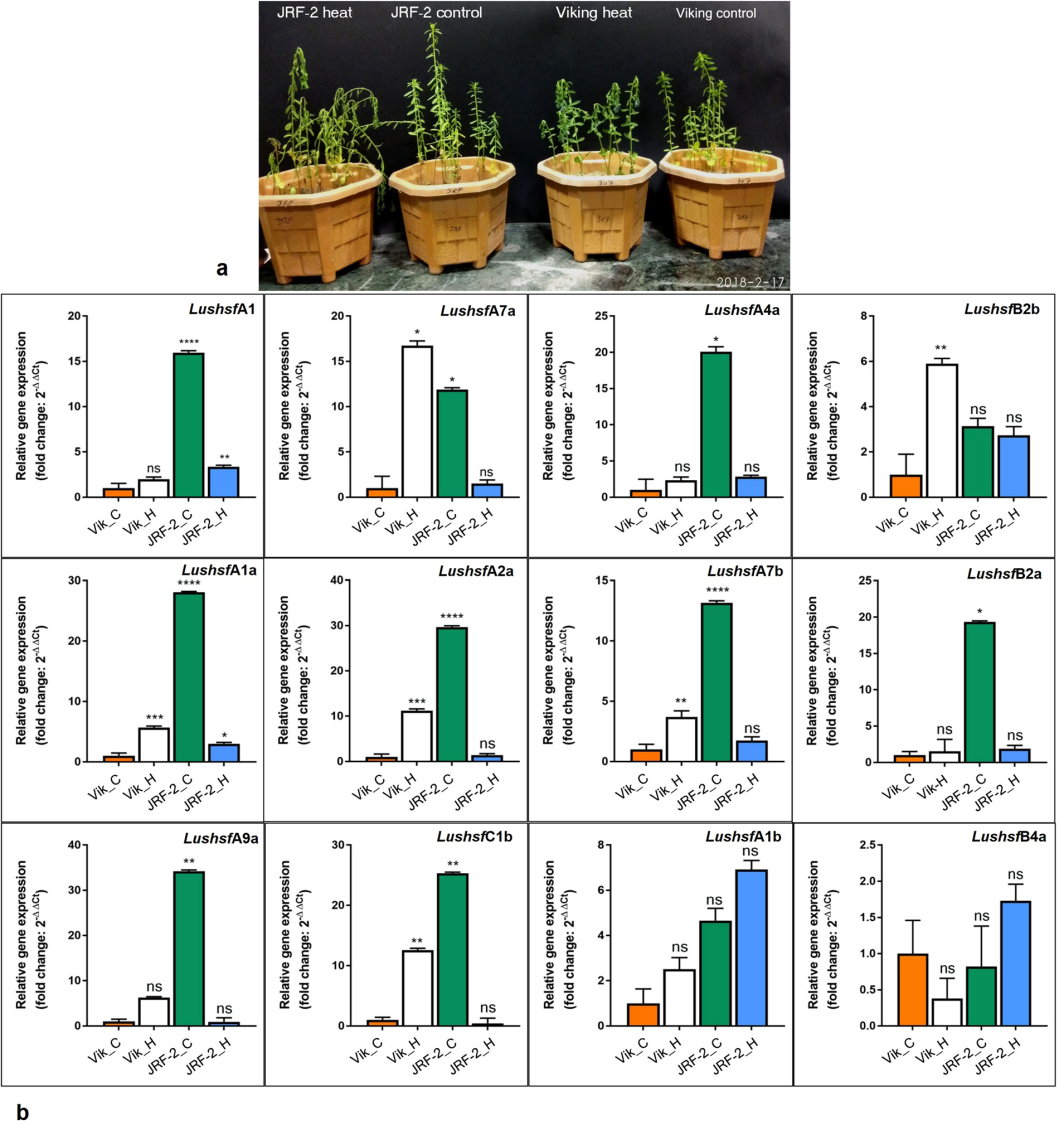
Relative quantification (RT-qPCR) of selective *LusHSF* genes between HT-treated and control plants of JRF-2 and Viking cultivars. (**a**) The upper panel shows the effect of HT stress treatments at 40 °C for 12 hrs in flax cultivars, which were used for total RNA extractions. (**b**) The relative gene expression fold change (2^−ΔΔ*Ct*^) of twelve selected *LusHSF* genes are represented as bar diagrams. The Ct values of each sample-*HSF* gene combination were normalized using the reference gene *ETIF3E* and calibrated with Ct values of Viking control to estimate (2^−ΔΔ*Ct*^) values. The statistical significance of the expression values is represented by ‘ns’ as non-significant, and ‘*‘ as significant at *p* > 0.05 and Bonferroni’s multiple comparisons test. One asterisk (*) represents adjusted *P* values between 0.01 and 0.05, and two asterisks (**) represent adjusted *P* values between 0.01 and 0.001, and so on. RNA samples include Vik_C: Viking under control conditions; Vik_H: Viking under HT stress conditions; JRF-2_C: JRF-2 under control conditions; JRF-2_H: JRF-2 under HT stress conditions.

We examined the expression pattern of the *LusHSF* genes under HT stress by comparing two different fibre flax cultivars, European Viking and Indian JRF-2, to measure the mRNA abundance in the shoot apex of 30 day-old control and HT-stressed (40 °C for 12 hrs) seedlings. From a preliminary screening, twelve *LusHSF* genes produced clear and consistent bands of expected size in both control and HT stressed samples. The remaining *LusHSF* genes either showed the presence/absence of bands or comprised non-specific amplicons (data not shown). The RT-qPCR analysis of the twelve *LusHSF* genes produced a differential expression pattern in the control and HT stressed plants (Fig. 8). Interestingly, in control JRF-2, the expression of a majority of the *LusHSF* genes was elevated when compared to that of the other samples (0.82 to 34.2-fold). In contrast, most of the *LusHSF* genes were down-regulated in the HT stress-treated JRF-2 (0.44 to 15.33) compared to those in the JRF-2 control plant. However, the *LushsfA1c* and *LushsfA1a*, were reasonably up-regulated in HT stress-exposed JRF-2. In HT-stressed Viking, the *LushsfA7a* and *LushsfB2b* genes were significantly up-regulated compared to those in control Viking and HT-stressed JRF-2 plants. Two genes, *LushsfA1b* and *LushsfB4a*, produced non-significant gene expression changes in all the samples compared to those in control Viking. Altogether, these differential expression patterns suggest their possible functional relevance in the HT stress response in a genotype-dependent manner.

### Prediction of CRISPR/Cas9 guide sequences with minimum off-target effects in the flax genome

We screened the *LusHSF* genes using an online CRISPOR tool to identify unique 20 bp *gRNA* sequences for each *LusHSF* gene. These *gRNA* sequences, which will serve as a resource for clustered regularly interspaced short palindromic repeats/CRISPR-associated 9 (CRISPR/Cas9)-based gene editing or functional studies, were compared and aligned to the *L*. *usitatissimum* genome. The *gRNA* sequences with the highest specificity and those located within 12 bp adjacent to the protospacer adjacent motif (PAM) sequence (the ‘seed region’) of the *gRNA* were considered for assessing minimum off-target effects. The total number of *gRNA* predictions for each *LusHSF* ranged from 74 to 223 with the 3′ PAM sequence NGG (where N = A/T/G/C). At a specificity score >50 (cutoff for high specificity), the number of *gRNA* sequences ranged from nine to 157. The *gRNA* sequences with the highest specificity score and minimum off-target effects are mentioned in Supplementary Table S3. Most of these *gRNA* sequences had the least off-target hits, ranging from 0 to 21 at the whole genome level, which may arise from 2 to 4 nucleotide mismatches. None of the *gRNA* sequences was predicted to produce off-target effects (up to ≤4 nucleotide mismatches) within the seed region, *i*.*e*., within the 12 bp adjacent to the PAM sequence. The forward and reverse primers were predicted for cloning and expression of all the *LusHSF* gRNA sequences using the T7 RNA polymerase-based system in the popular gene editing vector DR274 (Addgene plasmid # 42250). Specific restriction enzyme sequences were also predicted within the *gRNA* sequence at three bp 5′ to the PAM to facilitate the screening of mutation events induced by the *gRNA* in CRISPR experiments. Oligonucleotides with barcodes and corresponding sequencing primers to generate lentiviral saturation mutagenesis screens with the *LusHSF* genes, are also shown in Supplementary Table S3.

## Discussion

From past studies on HT stress in fibre flax, it is perceived that both low and HT stress, even in the absence of drought, are critical to flax growth and reproduction^7,8^. Seed germination, flowering, and seed setting in flax are optimum between temperatures of 16 °C to 25 °C. In a simulated experiment, HT during the initial growth phase, followed by a low temperature in the intermediate phase and HT during the late growth stages were observed as the most preferred conditions for fibre flax growth^11^. However, exposure to more than 40 °C for a stretch of five days during flowering in flax was detrimental, reducing seed yield and fibre quality^7^. Partial to complete necrosis of the ovules was the crucial limiting factor in poor seed setting due to HT stress in flax. A prolonged period of HT stress also forces the plant to undergo compensatory flowering^8^. This information warrants prioritized research on the genetic improvement of flax, especially fibre types, for terminal HT stress tolerance. In the long run, these findings will facilitate the acclimation of the superior fibre quality flax genotypes to a diverse climatic condition.

Among various genetic components, *HSFs* and *HSPs* play significant functions in responding to HT stress in plants. The former gene group plays the role of a regulatory partner in the functioning of the latter group, which serves as chaperones^12^. A fundamental knowledge of the role interplayed by these two key genetic factors is crucial beforehand to design a genetic improvement strategy for HT stress tolerance in any plant. Although the genome sequence of flax is available for the past few years^9^, the characterization of *HSFs* in flax has remained obscure until now. The present study involved the revelation of 34 true *HSF* sequences distributed in 14 out of the 15 flax chromosomes. A cumulative analysis of flax and other representatives from the order Malpighiales and commercial fibre crops, whose genome sequences are available, revealed a diverse *HSF* family size. Our report of 34 non-redundant complete *LusHSFs* is higher than those of *Ricinus* sp. (18) and *Corchorus* sp. (18), but lower than those of *Gossypium raimondii* (57), *Salix purpurea* (48), *P*. *trichocarpa* (47), and *M*. *esculenta* (39) (http://planttfdb.cbi.pku.edu.cn). Considering that whole genome duplication (WGD) events during ancient polyploidization and lineage-specific duplications are crucial factors for the speciation and expansion of gene family^13,14^, variations in the number of *HSFs* in flax and related plants have shed light on how this gene family has co-evolved. From WGD time estimates in Malpighiales, it is clear that flax has undergone two rounds of genome duplication: the earlier duplication occurring ~20–40 MYA and a more recent genome duplication at 5–9 MYA compared to the other plants analysed^15,16^. Intraspecies synteny analysis revealed that many of the *LusHSF* genes in flax genome constitute part of the syntenic blocks that still support their WGD origin. Genome duplication events simultaneously with gene gains or losses might have contributed to the diversity of the *HSF* gene family^17^. Through orthologue identification and d_*N*_/d_*S*_ substitution-based homology analysis of the *LusHSFs*, we could predict that the divergence time of *HSFs* in other related plant species occurred much earlier than those of flax, perhaps during ancient polyploidization event. Therefore, most of the putative *LusHSF* paralogues that co-evolved with the recent flax genome duplication event (5–9 MYA) might also correspond to diverse gene structures and functions.

In the present study, we describe a comprehensive characterization of the *LusHSF* genes and amino acid sequences to identify their important domains and motifs. All 34 selected LusHSF proteins comprised conserved characteristic domains, such as DBD, HR-A/B regions, NLS, NES, and CTAD; thus qualifying these proteins as true HSF proteins. Since the promoter regions are enriched with specialized *cis*-acting regulatory elements that also specify their putative functions^18^, we queried the promoter regions of the *LusHSFs*. The results revealed that the *LusHSF* promoters are enriched with a variety of regulatory elements related to abiotic stress tolerance, including the *HSE* and *LTRE*, which confer gene expression in response to high- and low-temperature conditions, respectively. From our digital gene expression analysis, using microarray data from the national center for biotechnology information (NCBI) database, evidence of the differential expression of the *LusHSF* genes was detected in different tissues. Transcriptional analysis of twelve *LusHSF* genes was also performed in two different fibre flax cultivars, Viking and JRF-2, under control and HT stress conditions. Interestingly, the analysis reveals that the abundance of the majority of the *LusHSF* mRNA is significantly higher in control JRF-2 (up to 34.2-fold) compared to that in control Viking. This difference may justify the better adaptability of the Indian JRF-2 cultivar under the hot and humid conditions of India compared to that of European Viking. In a few *LusHSFs*, a fold change in gene expression was also observed up to 16.74 times in the HT-stressed Viking and up to 15.33 times in the HT-stressed JRF-2. Overall, we noticed that the endogenous expression of *LusHSFs* in control JRF-2 was higher than that in the HT-stressed JRF-2. One possibility for the down-regulation of these *LusHSFs* in HT-stressed JRF-2 plants could be owing to prolonged HT stress treatment for over 12 hrs. A similar down-regulation of the *HSF* genes under HT shock treatment was recorded in plants^3^. All this information suggests that the *LusHSFs* might produce a differential response in different flax genotypes regarding HT stress and can be selected as candidate gene resources for functional studies and genetic improvements. Genetic engineering using candidate *HSF* genes was reported to impart enhanced thermotolerance to crop plants, such as in wheat^19^.

From our analysis of *LusHSFs*, we assume that these proteins are involved in differential functions in various tissues and under HT-stress induced responses. Since multifunctional HSF proteins have roles in various abiotic-stress tolerance responses^3,20^, the involvement of *LusHSFs* in regulating other traits cannot be ignored. Therefore, a comprehensive functional analysis of the *LusHSF* genes is a prerequisite before harnessing these molecules in any genetic improvement programme. For functional studies on *LusHSFs*, we were interested in determining the active macro-molecular binding sites on the LusHSF proteins. Computational predictions of the active sites for protein-DNA interactions can considerably reduce the cost and time of functional assays by providing a first-hand functional annotation. These computational predictions can be addressed using information from amino acid sequences or related protein structure models^21^. Through both the homology model and an amino acid sequence-guided approach, our analysis of the nucleoprotein interaction sites on the interface of LusHSFs aided us in identifying the active amino acid residues that might exert an effect on their functionalities, such as the HT stress response.

Genome editing using the CRISPR/Cas9 system is rapidly emerging as a tool for targeted gene knockout in plants, thus achieving functional analysis in a precise manner and in a rapid time^22,23^. Nevertheless, the success of CRISPR/Cas9 technology depends primarily on the specificity of the *gRNA* sequences designed to perform targeted gene-knockouts. CRISPOR is a simple web tool, that permits users to design *gRNA* for genome-wide CRISPR and saturation screens with information on the possible off-target effects on the genome of interest^24^. Our screen for *gRNA* sequences specific to the *LusHSF* genes produced sequences with minimum possible off-target effects, specifically within sequences adjacent to the PAM. This tool also predicted the *gRNA* cloning strategy for maximum functionality of the CRISPR system. Genome-wide analysis of a few important gene families, such as the genes controlling fatty acid biosynthesis^25^, chalcone synthase^26^, *β*-galactosidases^27^, cinnamyl alcohol dehydrogenase (CAD) genes^28^, NBS-LRR^29^, aquaporin^30^, pectinmethylesterases (PME) and pectinmethylesterase inhibitors (PMEI)^31^, UDP glycosyltransferase (UGT)^32^ and the dirigent protein family^33^ were carried out in the flax genome. However, designing the *gRNA* sequence for functional analysis of these gene families has not been attempted in any of these studies. Our present study, in addition to identifying active protein and DNA binding sites on LusHSF proteins, predicted and designed a number of *gRNA* sequences with the least off-target effects for functional analysis of the candidate *LusHSF* genes.

In summary, we identified 34 *LusHSF* genes with specific DNA and amino acid sequence features, plotted these genes on flax chromosomes and phylogenetically reconfirmed them into three broad groups and 13 subgroups based on their protein domains. The putative *LusHSF* paralogues were estimated as a recent gene duplication event than the orthologues in terms of their evolutionary gene family expansion. Functional predictions were based on various abiotic stress-related *cis*-acting elements detected in the promoter regions of the *LusHSFs*, the dynamics of digital gene expression patterns in different tissues, and the quantitative expression patterns of these genes under control and HT stress conditions. One of the key findings of the present study embodies the design of *gRNAs* for individual *LusHSFs* to promote further functional studies of this important gene family. However, a systematic analysis of gene expression under different temperatures and at different time intervals is imperative to assign a specific role to each candidate *LusHSF* gene before utilizing this gene resource in the genetic improvement of fibre flax for HT stress tolerance.

## Methods

### Retrieval and characterization of *HSF* sequences

Genomic, coding, protein and promoter sequences of the *HSF* gene family with conserved DBDs (Pfam ID: PF00447) from the *L*. *usitatissimum* (cv. CDC Bethune) genome were retrieved from the Phytozome database v12.1 (https://phytozome.jgi.doe.gov/pz/portal.html) using the BioMart tool. The protein sequences were confirmed using the batch search tool of the Hidden Markov Models (HMMs) in the Pfam 31.0 database^34^ with an E-value threshold of 10^−3^ and the Simple Modular Architecture Research Tool (SMART) for HSF DBD^35^. The putative *LusHSF* genes and their detailed classifications were further identified against the HEATSTER platform^20^ (http://www.cibiv.at/services/hsf/) and MARCOIL (http://toolkit.tuebingen.mpg.de/marcoil) to determine the presence of coiled-coil structures. The isoelectric point (pI) and molecular weight of LusHSF proteins were estimated from the Compute pI/Mw tool of Expassy^36^ (http://www.expasy.org/). The grand average of hydropathy (GRAVY) scores, which is based on the hydropathy of all the amino acids of a protein molecule and determines whether a protein is polar or non-polar in nature^37^, were estimated using the GRAVY calculator (http://www.gravy-calculator.de/). Protein subcellular localization of the LusHSFs were predicted by using WoLF PSORT^38^ (http://www.genscript.com/wolf-psort.html).

### Chromosomal mapping and analysis of gene duplication

The genomic coordinates of the 34 *LusHSF* genes were mapped on flax chromosome^10^ using the software Graphical Geno Typing v2.0 (GGT 2.0)^39^ and MapChart v2.3^40^. The paralogous relationships of the *LusHSF* genes were identified according to their duplication patterns (tandem or block) using conditional reciprocal blast (crb-blast)^41^ with a stringent E-value of 1.0e^−50^. The paralogous partners were identified based on query coverage >90% and percentage of identical matches >70%. Patterns of genome duplication among the putative *LusHSF* paralogues and their adjacent genomic regions were analysed using the GEvo tool of the CoGe database (https://genomevolution.org/coge/) and the (B)LastZ: Large Regions algorithm^42^.

### Multiple sequence alignments, phylogeny, and classification

The amino acid sequences of the conserved DBD and OD with HR-A/B motifs of LusHSFs were deduced from the HEATSTER platform^20,43^ for multiple sequence alignments using the online Clustal Omega tool of EMBL-EBI^44^ (http://www.ebi.ac.uk/Tools/msa/clustalo/) and visualized using BoxShade v3.21 (http://www.ch.embnet.org/software/BOX_form.html). For phylogenetic tree reconstruction and reconfirming the classification of the LusHSFs, the amino acid sequences from the start of DBD to the end of OD domains of flax, Arabidopsis (dicot model plant), and rice (monocot) HSFs were retrieved for multiple sequence alignments. The alignment was performed using MUSCLE algorithm and 16 maximum iterations in the MEGA-X software^45^. The phylogenetic tree was inferred by using the Maximum Likelihood (ML) method and the Jones-Taylor-Thornton (JTT) matrix-based amino acid substitution model^46^. The best model was estimated using the model selection tool in MEGA-X and from the lowest Bayesian Information Criterion (BIC) score. A discrete gamma distribution was chosen to model evolutionary rate differences among sites [16 categories (+G, parameter = 1.6109)]. The initial tree for the heuristic search were obtained automatically by applying Neighbor-Join and BioNJ algorithms to a matrix of pairwise distances estimated using a JTT model, and then selecting the topology with superior log likelihood value. All positions with less than 95% site coverage were eliminated, *i*.*e*., fewer than 5% alignment gaps, missing data, and ambiguous sequences were allowed at any position (partial deletion option). The test of phylogeny was conducted using 1000 bootstrap replications. All other parameters of phylogenetic tree reconstruction were kept default. Using similar parameters, a phylogenetic ML tree was also reconstructed using the HSFs from closely related sequenced plants of the order Malpighiales and commercial fibre crops, like cotton and *Corchorus* spp.

### Gene structures, protein domain distributions and DNA-binding site predictions

The exon/intron and splicing phase in *LusHSF* genes were derived by aligning the corresponding CDS and genome FASTA sequences in the Gene Structure Display Server (GSDS2.0) (gsds.cbi.pku.edu.cn/) programme^47^. The LusHSF DBD coordinates on the protein and the phylogenetic tree in Newick format were used as inputs to display the gene structures. The distribution of protein domains, such as DBD, OD (HR-A/B), NLS and NES, on the LusHSF amino acid sequences were determined from the online HSF prediction tool of the HEATSTER platform^20,43^, and Interproscan^48^. The conserved domains and motifs were visualized using the Illustrator for Biological Sciences (IBS) v.1.0.3^49^ (http://ibs.biocuckoo.org/). Additionally, the LusHSF protein sequences were scanned for the prediction of protein-protein and protein-DNA binding interface identification using a FASTA sequence search approach in the online open PredictProtein server^50^ (https://open.predictprotein.org/) and comparative model-based TFmodeller web server^51^ (http://maya.ccg.unam.mx/$\sim$tfmodell/).

### Orthologue identification, synteny mapping, and evolutionary analysis

Putative orthologues of *LusHSF* genes were identified from *Arabidopsis*, poplar (*P*. *trichocarpa*), castor bean (*R*. *communis*), cassava (*M*. *esculenta*), soybean (*G*. *max*), and grape (*V*. *vinifera*) *HSFs*, which were derived from the plant transcription factor database v.3.0 (PlantTFDB)^52^ (http://planttfdb.cbi.pku.edu.cn/index.php). The crb-blast program^41^ at an E-value of 1.0e^−50^ was employed for this purpose. The top query and subject BLAST hits were filtered using >70% identity and >90% query and subject coverage in the Microsoft Excel program. Orthologues were also inferred using the OrthoFinder v.1.1.8^53^ and compared with crb-blast output. Only the consistent putative orthologues were used for synteny mapping. The corresponding genomic coordinates of the putative orthologous gene pairs were derived from the respective genomes in the Phytozome database v.12.1, and the orthologous relationships were visualized using CIRCOS v0.69-5^54^. The d_*N*_/d_*S*_ estimation of putative *LusHSF* homologue sequences (both orthologues and paralogues) was conducted using PAL2NAL (http://www.bork.embl.de/pal2nal/) in the codeml program in PAML^55^. The evolutionary time (T) or likely gene duplication event of the *HSF* genes was calculated in terms of million years ago (MYA) using a synonymous mutation rate of *λ* substitutions per synonymous site per year. A d_*N*_/d_*S*_ ratio <1, >1, and = 1 indicates negative (purifying selection), positive, and neutral evolution, respectively.

### *Cis*-acting regulatory element identifications

To predict the putative promoter region based on the transcription start site of plants (TSSP) in 1000 bp sequences upstream of *LusHSF* genes, the TSSP online program of SoftBerry (http://www.softberry.com/berry.phtml) was used. The unverified string of bases from the putative promoters was removed from the analysis. The *cis*-acting regulatory elements were searched on the putative promoter regions of *LusHSF* genes using plant *cis*-acting regulatory DNA elements (PLACE; https://www.hsls.pitt.edu/obrc/index.php?page=URL1100876009) and plant *cis*-acting regulatory elements (PlantCARE; http://bioinformatics.psb.ugent.be/webtools/plantcare/html/) databases^56,57^. Both positive and negative promoter DNA strands were subjected to a *cis*-element search. Only the abiotic stress-related regulatory elements were retrieved.

### Digital gene expression analysis

Gene expression data in terms of microarray and transcriptome sequences from the different flax tissues and developmental stages of the NCBI Gene Expression Omnibus (GEO) repository were downloaded to analyse the digital gene expression of *LusHSF* genes. Microarray data (accession number GSE21868), from inner- and outer-stems, embryo, leaves, and roots^58^ were subjected to a homology-based (blastn) similarity search with an E-value cutoff of 1.0e^−50^ to *LusHSF* sequences. The best *LusHSF*-aligned unigene sequences (≥95% identity) were considered to derive log_2_ values from the microarray data in robust multi-array average (RMA) values. The mean log_2_ values for different tissues are represented by a heatmap diagram. Similarly, other microarray data, such as GSE61311 (unpublished), with inner and outer stem tissues from a wild-type and its mutant (*lignified bast fibre mutant* 1) plants, and the RNA-seq data for the flax shoot apex (GSE80718)^59^, were searched, and corresponding fragments per kilobase of transcript per million mapped read (FPKM) values were log_2_ transformed before plotting in heatmaps. All heatmaps were generated using the Heatmap Illustrator (HemI v.1.0)^60^ and clustering was performed using the hierarchical method, average linkage, and Euclidean distance similarity metric.

### Plant samples, HT stress treatment, and RT-qPCR analysis

The seeds of two different winter fibre flax cultivars, European Viking and Indian JRF-2 (Tiara), were grown under controlled glass-house conditions. The former cultivar was a French introduction, while the latter was a released variety for the conditions in India. Our initial field observation showed Viking as a heat-susceptible cultivar (deformed inflorescence, poor flowering, and seed setting) compared to JRF-2. Total RNA was extracted from the shoot apex tissues of 30-day-old control and HT stressed (40 °C for 12 hrs in a plant growth chamber) flax seedlings using TRIzol reagent (Invitrogen, Thermo Fisher Scientific, Inc., USA) according to the manufacturer’s instructions. Approximately, 5 *μ*g/mL of DNaseI-treated total RNA was reverse transcribed using the SuperScript III First Strand cDNA synthesis system (Invitrogen Inc., USA) to generate cDNA according to the manufacturer’s protocol. Gene-specific (*LusHSF*) and a reference gene *eukaryotic translation initiation factor* (*ETIF3E*) primers^61^ were designed using the Quant Prime^62^ tool and synthesized at Eurofins Genomics India Private Limited, India (Supplementary Table S4). The RT-qPCR analysis was performed using PowerUpTM SYBR Green Master Mix (Applied Biosystems, Inc., USA) on a CFX Connect Real-Time PCR Detection System (Bio-Rad, Inc., USA). Each qPCR reaction (20 *μ*L) consisted of 10 *μ*L SYBR-Green mix, 4 *μ*L cDNA template (120 ng), and 1.0 *μ*L of 10 *μ*M solution of each forward and reverse primers. The PCR cycling programme consisted of 50 °C for 2 min, 95 °C for 5 min followed by 40 cycles at 94 °C for 10 s, 55 °C for 20 s, and 68 °C for 30 s. To analyse the specificity of the amplicons, a melting curve analysis was performed at 95 °C for 30 s, 65 °C for 30 s, followed by ramping up to 95 °C with 0.5 °C increment per cycle. For each sample, three technical replicates were conducted to minimize the PCR artefacts. The relative expression of each selected gene was averaged from the differences in cycle threshold (Ct) values normalized against the reference gene and finally calibrated against the control RNA sample from the Viking accession. The relative quantification method (2^−ΔΔ*Ct*^)^63^ was plotted as fold change gene expression in all the samples utilizing the GraphPad Prism software trial version (https://www.graphpad.com/scientific-software/prism/). One-way ANOVA, followed by Bonferroni’s multiple comparisons test correction was employed to analyse the statistical hypothesis at *p* = 0.05.

### Predictions of guide RNA sequences for gene editing and off-target effects

The web server CRISPOR v4.4^24^ (http://crispor.tefor.net/) was employed to predict efficient *gRNA* sequences for CRISPR/Cas9-based gene editing experiments in *LusHSFs*. The genomic DNA sequences of *LusHSFs* were scanned as input sequences for the identification of unique 20 bp target *gRNA* sequences against the *L*. *usitatissimum* genome of Phytozome v.9. *LusHSF* genes with >2000 bp lengths were scanned up to <2000 bp from the 5′ translational start site as per the requirement of the tool. To facilitate the use of the popular CRISPR/Cas9 system and employ *Streptococcus pyogenes* Cas9 nuclease, the corresponding *NGG* trinucleotide was selected as the protospacer adjacent motif (PAM). The *gRNA* sequences for the respective *LusHSFs* were chosen based on the highest specificity score and least probable off-target cleavage sites, especially within the 12 bp region adjacent to the PAM, known as the ‘seed region’. The *gRNA* sequences are allowed at least four nucleotide mismatches for the probability of off-target effect predictions. Oligonucleotides for lentiviral saturation mutagenesis screening were also identified along with specific barcodes. In a saturating mutagenesis experiment, a target region of the genome is altered with many guides, to create as many DNA edits as possible followed by mutant phenotyping. The corresponding Illumina sequencing primers were also designed for each *LusHSF* with the Illumina adapters TCGTCGGCAGCGTCAGATGTGTATAAGAGACAG and GTCTCGTGGGCTCGGAGATGTGTATAAGAGACAG to validate the gene sequence modifications.

## Supplementary information

Supplementary Figures S1, S2, S3 and S4

Supplementary Tables S1, S2, S3 and S4

## Data Availability

All data generated or analysed in the study are included in this article or in its supplementary information files.
