# Supplementary material for: Genomic insights into HSFs as candidate genes for high-temperature stress adaptation and gene editing with minimal off-target effects in flax
Source: Sci Rep. 2019 Apr 3;9:5581. doi: 10.1038/s41598-019-41936-1 (PMC6447620; doi:10.1038/s41598-019-41936-1)
Supplement: Supplementary file 1 — Supplementary Figures S1, S2, S3 and S4 [file 41598_2019_41936_MOESM1_ESM.pdf]

Supplementary Figure files

Genomic insights into *HSF*s as candidate genes for high-temperature stress adaptation and gene editing with minimal off-target effects in flax

Dipnarayan Saha[1, *], Pranit Mukherjee[1], Sourav Dutta[1], Kanti Meena[1], Surja Kumar Sarkar[1], Asit Baran Mandal[1], Tapash Das Gupta[2] & Jiban Mitra[1]

Affiliations

[1]Division of Crop Improvement, ICAR-Central Research Institute for Jute and Allied Fibres, Kolkata, West Bengal India 700121

[2]Faculty of Integrated Rural Development and Management, Ramakrishna Mission Vivekananda Educational and Research Institute, Ramakrishna Mission Ashrama, Narendrapur, Kolkata 700103, West Bengal, India

*Corresponding author Dipnarayan Saha: dipnarayan.saha@icar.gov.in; dipsaha72@yahoo.com; Orcid ID: https://orcid.org/0000-0002-5809-4340

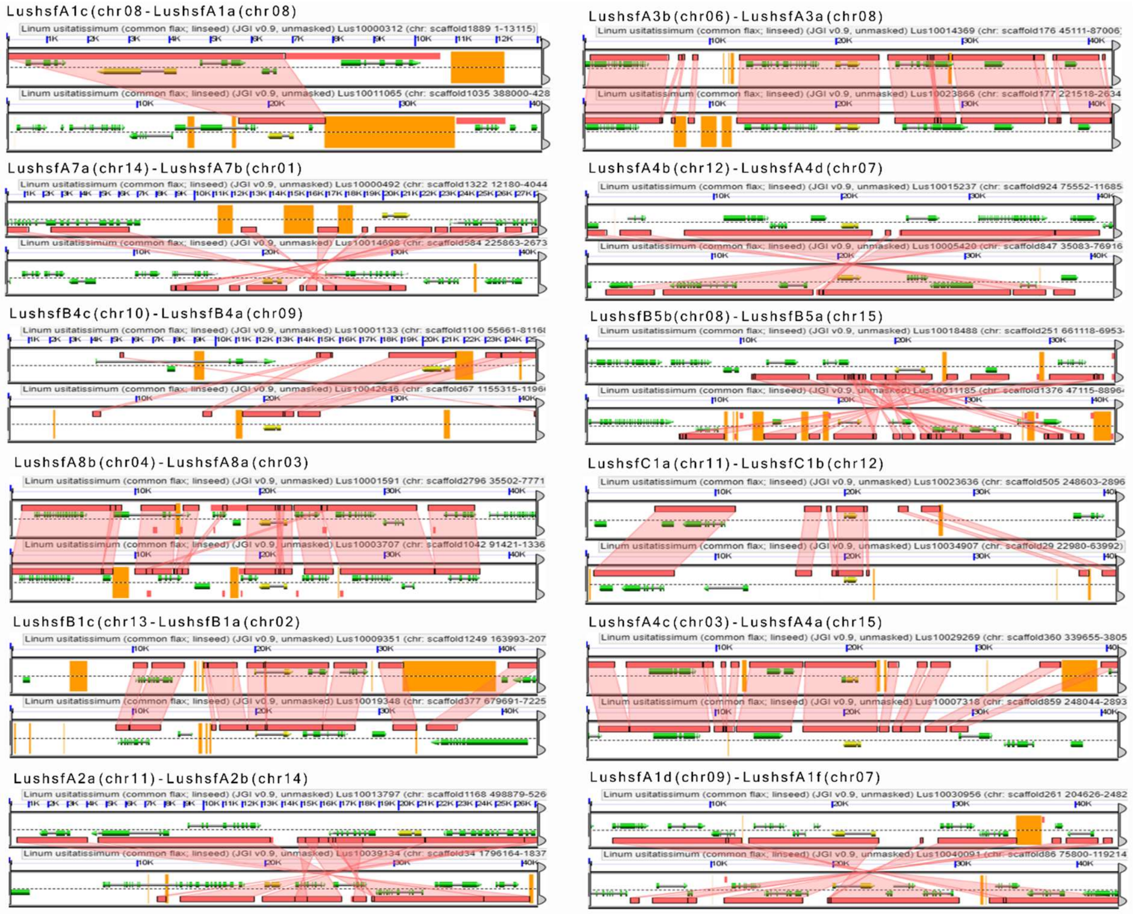

Supplementary Figure S1. GEvo (CoGe) microsynteny analysis of *LusHSF* gene paralogs and its adjacent genomic regions. Each pair of paralogous *LusHSF* gene-pairs are denoted as golden bars. Microsyntenic regions are shaded in pink. Inverted microsyntenic regions possibly arose due to genomic inversion. Regions with orange shades represent poor sequence quality and chromosomal breakpoints.

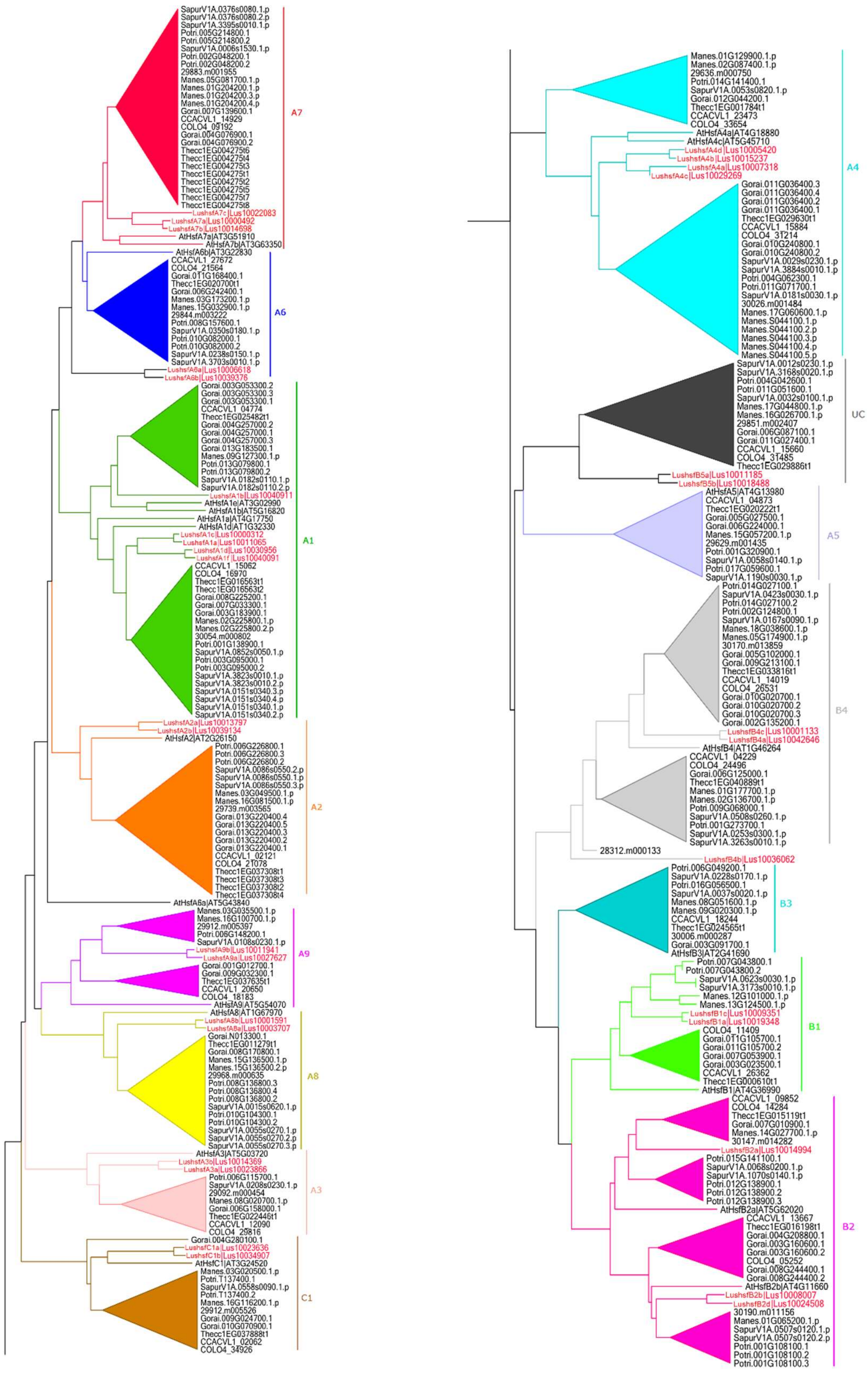

Supplementary Figure S2. Phylogenetic relationship of *LusHSF* genes to *HSFs* from closely related sequenced plants of order Malpighiales order and other commercial fibre crops. The tree was generated using FigTree v1.4.2. Position of *LusHSF* genes are marked in red. The closely clustered *HSF* genes supported with high boot strap values are marked with cartoon triangles. Phylogenetic groups are mentioned on the right.

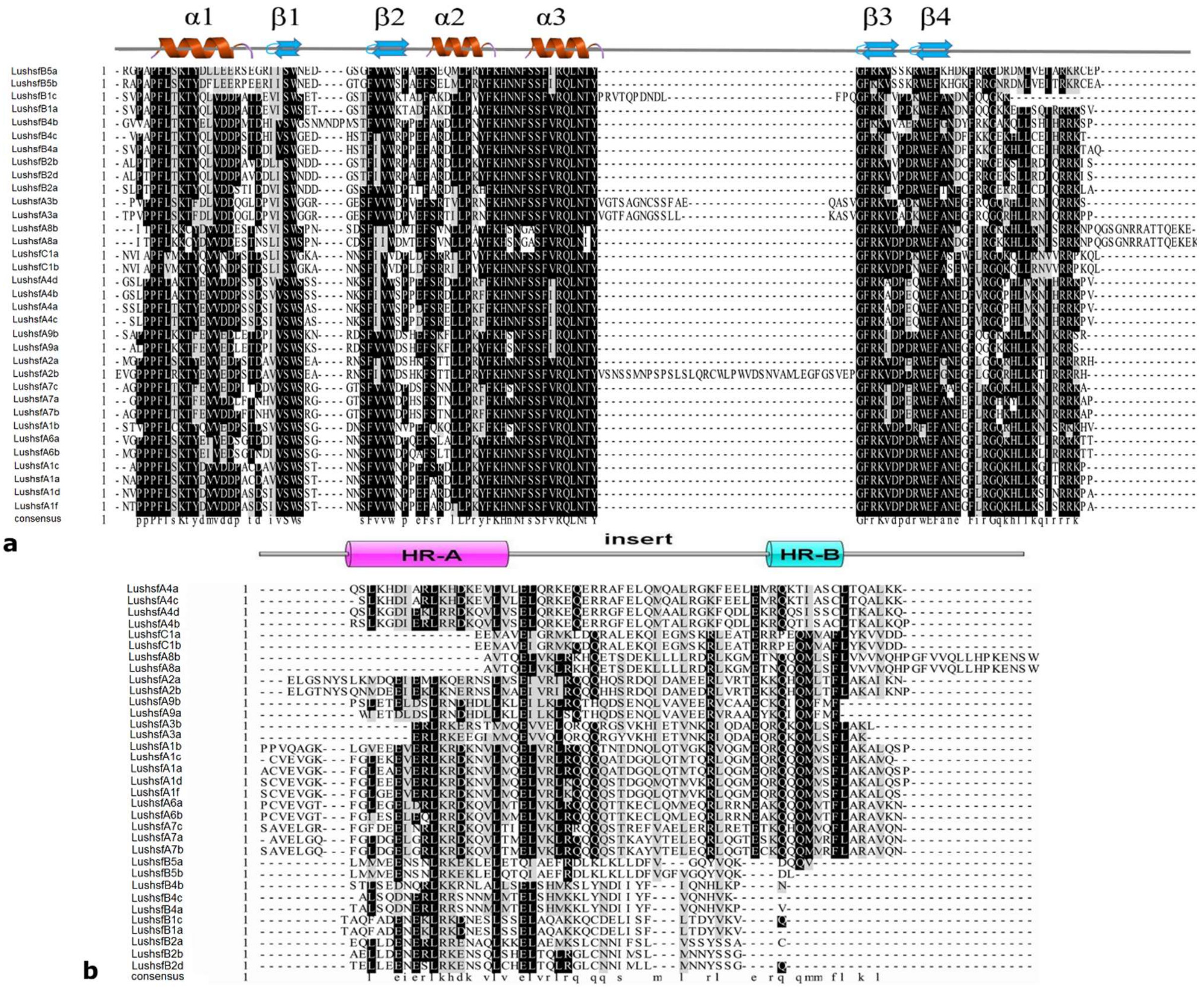

Supplementary Figure S3. Multiple sequence alignment (MSA) of amino acid residues of conserved (a) DBD and (b) HR-A/B motifs of LusHSF proteins and visualized using BoxShade v3.21. The positions of α helix and β pleated sheets were denoted on top of the Fig. **a**. The N-terminal motif HR-A, C-terminal motif HR-B, and the insertion residues were mentioned on top of the Fig. **b**. Consensus amino acid residues of the MSA was presented in bottom of the Fig. **a** & **b**.

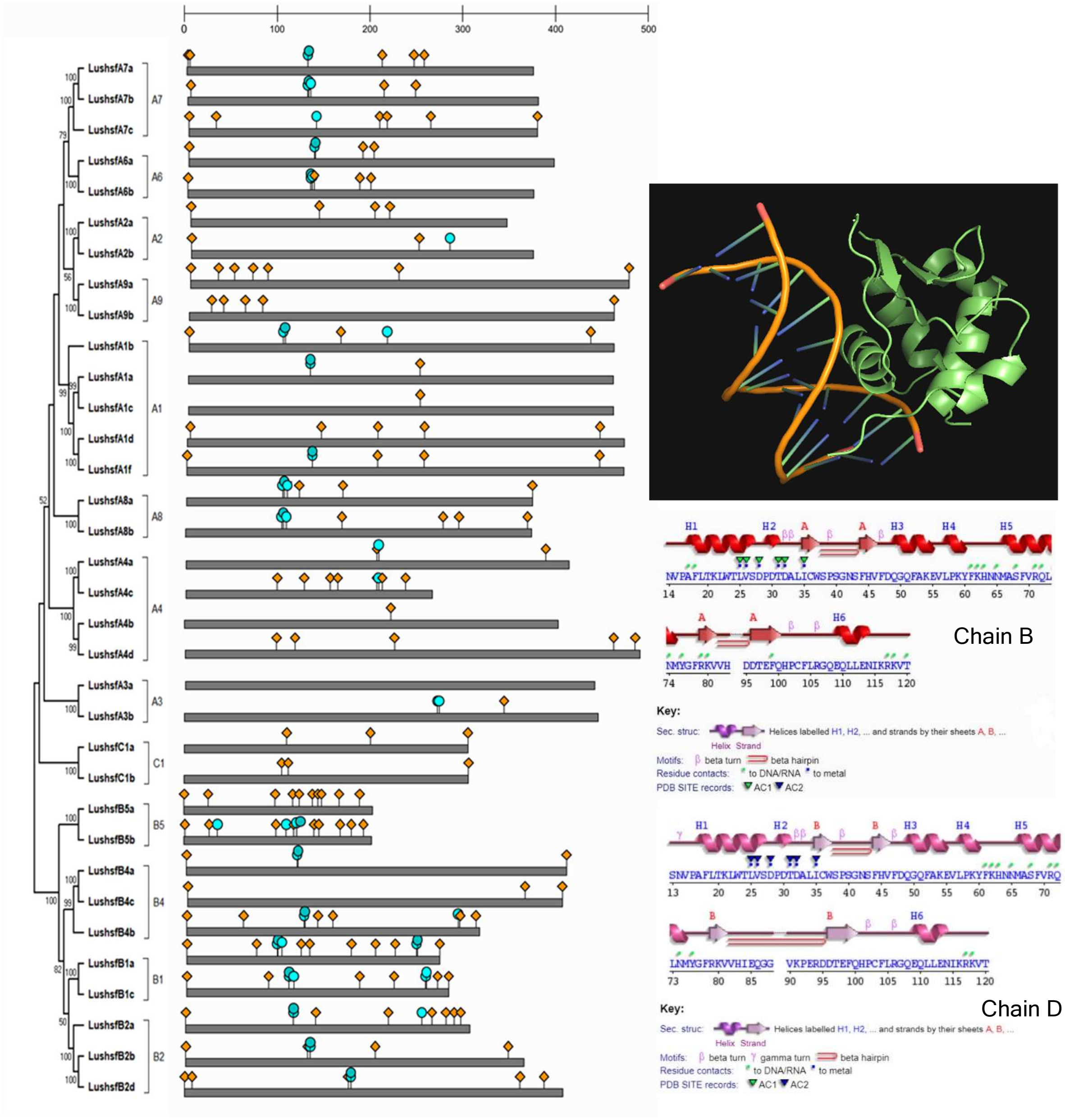

a b

Supplementary Fig. S4. (a) Position of active amino acid residues predicted for binding of proteins and polynucleotides on LusHSF protein sequences. The active binding sites were predicted using PredictProtein open server (https://open.predictprotein.org/) and the diagrams were generated using Illustrator for Biological Sciences (IBS) v.1.0.3. **(b) PDB template of human heat-shock transcription factor model 5d5v_B used to identify DNA contact interfaces on the LusHSF proteins.** The model was visualized using the open source PyMOL molecular graphics system (https://sourceforge.net/projects/pymol/?source=navbar). Secondary structures of B and D chain with DNA binding sites generated using PDBsum (http://www.ebi.ac.uk/thornton-srv/databases/cgi-bin/pdbsum/GetPage.pl?pdbcode=index.html) are also shown below.
